# Preventative Cancer Vaccine-Elicited Human Anti-MUC1 Antibodies Have Multiple Effector Functions

**DOI:** 10.3390/antib13040085

**Published:** 2024-10-10

**Authors:** Michelle L. McKeague, Jason Lohmueller, Matthew T. Dracz, Najla Saadallah, Eric D. Ricci, Donella M. Beckwith, Ramya Ayyalasomayajula, Maré Cudic, Olivera J. Finn

**Affiliations:** 1 Department of Immunology, University of Pittsburgh, Pittsburgh, PA 15213, USA; lohmuellerj@upmc.edu (J.L.); ojfinn@pitt.edu (O.J.F.); 2 Division of Surgical Oncology, Department of Surgery, University of Pittsburgh, Pittsburgh, PA 15213, USA; 3 Center for Systems Immunology, University of Pittsburgh, Pittsburgh, PA 15213, USA; 4 UPMC Hillman Cancer Center, University of Pittsburgh, Pittsburgh, PA 15213, USA; 5 Department of Psychology, Dietrich College of Humanities and Social Sciences, Carnegie Mellon University, Pittsburgh, PA 15213, USA; 6 Department of Chemistry and Biochemistry, Florida Atlantic University, Boca Raton, FL 33431, USA

**Keywords:** mucin-1, phagocytosis, trogocytosis, NK cell, monocyte, neutrophil, tumor, vaccine, epitope properties, O-glycosylation

## Abstract

Background/Objectives: Mucin-1 (MUC1) is a transmembrane glycoprotein that is overexpressed and hypoglycosylated in premalignant and malignant epithelial cells compared to normal cells, creating a target antigen for humoral and cellular immunity. Healthy individuals with a history of advanced colonic adenomas and at high risk for colon cancer were enrolled in a clinical trial to evaluate the feasibility of using a MUC1 peptide vaccine to prevent colon cancer. Anti-MUC1 antibodies elicited by this vaccine were cloned using peripheral blood B cells and sera collected two weeks after a one-year booster. Twelve of these fully human monoclonal antibodies (mAb) were tested for binding to MUC1+ target cells, and three with the highest binding were further evaluated for various effector functions important for tumor rejection. Methods: Immune cells were incubated together with target cells expressing variations in the number, distance, and membrane anchoring properties of the MUC1 epitope in the presence of each mAb. Results: All three mAbs mediated antibody-dependent cytokine release (ADCR), antibody-dependent cellular cytotoxicity (ADCC), and antibody-dependent cellular phagocytosis (ADCP). Two also mediated antibody-dependent trogocytosis/trogoptosis (ADCT). None were capable of complement-dependent cytotoxicity (CDC). Conclusions: ADCP and ADCT functions were more efficient when antibodies bound epitopes proximal to and anchored to the membrane, providing insight for future therapeutic antibody validation strategies.

## 1. Introduction

The capacity of the human immune system to recognize and subsequently eliminate malignant cells is the underlying principle of the expanding field of cancer immunotherapy. That it can also recognize and eliminate premalignant cells is the basis for the field of cancer immunoprevention. One change that occurs early in premalignant cells is a change in expression of the cell surface glycoprotein MUC1, or mucin-1. MUC1 is a large, 250 to 500 kDa variable-number tandem repeat (VNTR)-containing transmembrane protein comprised of two non-covalently associated subunits that is densely O-glycosylated on the apical surface of healthy cells, including breast, pancreatic, colonic, ovarian, gastric, and lung tissue [1]. Each individual can express copies of MUC1 with between 25 and 125 repeats of the identical 20-amino acid long sequence HGV**TS**APD**T**RPAPG**ST**APPA, each containing 5 sites for O-glycosylation post-translational modifications at serine and threonine residues [2]. When cells begin to transition into premalignancy, MUC1 loses its apical polarity and becomes overexpressed and hypoglycosylated due to glycosylation enzyme and chaperone molecule expression changes [3,4,5,6]. The hypoglycosylated tumor form of MUC1 is found on many adenocarcinomas, including those of the breast, prostate, lung, ovaries, pancreas, colon, and stomach, as well as certain hematopoietic malignancies such as T and B cell lymphomas, leukemias, and multiple myelomas [7,8]. Fewer and less branched sugars, including the tumor-associated carbohydrate antigens (TACAs) *N*-acetylgalactosamine (GalNAc), also known as Thomsen Nouveau (Tn, CD175), sialyl Tn (CD175s), Thomsen-Friedenreich (TF, CD176, T antigen), and sialyl-TF, reveal the normally masked MUC1 peptide backbone and aberrant glycoepitopes, to which both cellular and humoral immune responses can be generated [9]. The presence of naturally occurring anti-MUC1 antibodies specific for the unglycosylated peptide backbone epitopes has been correlated with better disease prognosis and serves as a well-established biomarker for multiple cancer types [10,11].

Various unglycosylated or hypoglycosylated (tumor) forms of MUC1 have been tested as therapeutic vaccines in patients with advanced cancer [12]. More recently, unglycosylated MUC1 peptide was used in a cancer prevention setting to vaccinate healthy individuals with a history of colonic polyps who were at high risk for developing colon cancer [13]. Fully human MUC1-specific antibodies were cloned from a vaccinated participant into an IgG1 backbone vector and evaluated for their ability to recognize MUC1 on tumor cells [14]. In this study, we further characterize 12 of these antibodies for binding to MUC1 on target cells and fully elucidate the mechanisms of action by which 3 of these antibodies might eliminate or facilitate elimination of tumor cells. As MUC1 is a tumor target with complex attributes, including a very large size and numerous tandemly repeated epitopes that could be located both near and far from the tumor cell surface [15], we also aimed to better understand how various properties of the antigen affect anti-tumor antibody efficacy. To this end, we co-cultured immune effector cells, anti-MUC1 antibodies, and target tumor cells while varying attributes of their MUC1 antigen, such as glycosylation, epitope distance from the membrane, total number of epitopes per molecule and per cell, and attachment of the extracellular domain of the molecule to the cell surface.

## 2. Materials and Methods

### 2.1. Antibodies

Fully human antibodies were isolated from serum and peripheral blood mononuclear cells (PBMC) from a participant in the clinical trial NCT00773097 [13], and as previously described [14], their variable region sequences were cloned into plasmids containing an IgG1 constant region and expressed in HEK293 cells (Cell Signaling Technology, Danvers, MA, USA). Herceptin (trastuzumab, Genentech, South San Francisco, CA, USA) and Rituxan (rituximab, Genentech, South San Francisco, CA, USA) were both reconstituted from clinical-grade lyophilized preparations. The murine anti-MUC1 mAbs, anti-CD227 (HMPV)-FITC (BD Biosciences, San Jose, CA, USA), and 3C6, a gift from the late Dr. Hilgers (Free University, Amsterdam, The Netherlands), together with an APC-Cy7-conjugated anti-mouse IgG secondary (eBioscience, San Diego, CA, USA), were used to stain MUC1 on target cells. APC-conjugated F(ab’)2 fragments specific to human IgG (Jackson Immunoresearch, West Grove, PA, USA) or Alexa488-conjugated goat anti-human IgG (Invitrogen, Carlsbad, CA, USA) were used as secondary reagents to detect the unlabeled human IgG primary antibodies.

### 2.2. Cell Lines

Raji B cell lymphoma (CCL-86) cells, Jurkat T cell leukemia Clone E6–1 (TIB-152) cells and THP-1 monocyte cells (TIB-202) were obtained from the American Type Culture Collection (ATCC, Manassas, VA, USA). Cells were cultured at 37 °C in complete RPMI medium containing 10% fetal bovine serum, 1% L-glutamine, 1% sodium pyruvate, 1% Penicillin-Streptomycin, and 1% non-essential amino acids.

### 2.3. Primary Cells

Healthy donor white blood cells were isolated from de-identified whole blood samples or buffy coat samples purchased from the Pittsburgh Central Blood Bank, fulfilling the basic exempt criteria 45 CFR 46.101(b)(4) in accordance with the University of Pittsburgh IRB guidelines. Lymphocyte Separation Medium (LSM, MP Biomedicals, Irvine, CA, USA) density gradients were used to isolate PBMCs away from granulocytes. Red blood cells were lysed with ACK lysis buffer.

### 2.4. Lentiviral Vector Generation

DNA constructs for the coding regions listed in Table S1 were first synthesized (Integrated DNA Technologies, Coralville, IA, USA) and then cloned into the pSICO transfer vector backbone containing the EF1-alpha promoter, pSICO-EF1 (Addgene Plasmid #21375), or the pHR_PGK vector containing the PGK promoter (a gift from Wendell Lim, Addgene Plasmid #79120), using Gibson Assembly cloning as previously described [14].

### 2.5. Lentiviral Production

To generate each virus, HEK293T cells (ATCC, CRL-3216) were transfected with the packaging constructs pVSV-G (VSV glycoprotein expression plasmid), pLP2 (Rev expression plasmid), and pLP1 (Gag/Pol expression plasmid), along with a pSICO-EF1-MUC1 or pHR_PGK transfer plasmid using calcium phosphate transfection. After 16 h post-transfection, cells were first washed with PBS and fresh, complete DMEM medium containing 6 mM sodium butyrate was added (Sigma Aldrich, St. Louis, MO, USA). After 24 h, the viral supernatants were collected, and cells were again given fresh media with sodium butyrate. Additional supernatants were collected 24 h after the first collection, and both sets of supernatants were combined and filtered through a 0.45 μm vacuum filter. Viral particles were concentrated by ultracentrifugation for 1.5 h at 24,500 rpm, and the viral pellets were re-suspended in 50 µL of complete RPMI medium and stored at −80 °C.

### 2.6. Lentiviral Transduction

Frozen lentivirus was thawed for 10 min at room temperature and added to cells at a multiplicity of infection (MOI) of 10–50 in the presence of 6 mg/mL DEAE-dextran (Sigma Aldrich, St. Louis, MO, USA) in complete RPMI. Cells were spun down, and culture medium was replaced the next day.

### 2.7. Cell Sorting

Transduced cells were harvested, stained with anti-CD227 (HMPV)-FITC (BD Biosciences, San Jose, CA, USA), filtered, and sorted on a BD FACSAria instrument.

### 2.8. Antibody Binding Assay

Transduced cells stably expressing human MUC1 were stained with Ghost Dye Red 780 (Tonbo Biosciences, San Diego, CA, USA) or 7-AAD (STEMCELL Technologies, Vancouver, BC, USA) to measure viability and 10 μg/mL anti-human MUC1 mAbs for 10 min at 37 °C or between 10 to 30 min at room temperature. Cells were washed and stained with fluorescently conjugated anti-human IgG secondary antibodies for 10 min at room temperature. Following some of the functional assays, cells were washed, stained with Ghost Dye Red 780 to measure viability, and then incubated with fluorescently conjugated anti-human IgG secondary antibodies for 10 min at room temperature to measure antibody binding levels that were present on live cells throughout the duration of the assays (Figure S1). Cells were analyzed on an LSRFortessa cytometer (BD Biosciences, San Jose, CA, USA).

### 2.9. Antibody-Dependent Cellular Cytotoxicity Assay

Experiments were performed with target cell lines and magnetically-enriched healthy donor NK cells as previously described [16].

### 2.10. Antibody-Dependent Cytokine Release Assay

Healthy donor PBMCs were incubated for 4 h with target cells at an effector to target ratio (E:T) of 25:1 and human mAbs (10 µg/mL) and supernatants were collected, stored at −80 °C, and assayed for the presence of 13 cytokines in the LEGENDplex Human Inflammation panel 1: IL-1β, IFN-α2, IFN-γ, TNF-α, MCP-1, IL-4, IL-6, IL-8, IL-10, IL-12p70, IL-15, IL-17A, Il-18, IL-23, and IL-33 (LEGENDplex™, BioLegend, San Diego, CA, USA). Serial dilutions of cytokine standards present in the kit were included on each plate. Samples were analyzed on an LSRFortessa cytometer (BD Biosciences, San Jose, CA, USA). Cytokines at 2 pg/mL or below were considered undetectable.

### 2.11. Antibody-Dependent Cellular Phagocytosis Assay

Target cells were labeled with 1 µM CellTrace Yellow (Invitrogen, Carlsbad, CA, USA) and effector THP-1 cells with 1 µM CellTrace Violet (Invitrogen, Carlsbad, CA, USA), and cells were stained with 10 µg/mL human IgG antibodies prior to 1 h of co-incubation at 37 °C, as previously described [16]. Cells were stained with Ghost Dye Red 780 (Tonbo Biosciences, San Diego, CA, USA) to measure viability, washed, and resuspended for analysis on an LSRFortessa (BD Biosciences, San Jose, CA, USA).

### 2.12. Antibody-Dependent Trogocytosis/Trogoptosis Assay

Similar to the protocol described by Matlung et al. [17], healthy donor human neutrophils were stimulated overnight with 10 ng/mL G-CSF (Peprotech, Cranbury, NJ, USA) and 50 ng/mL IFNγ (R&D Systems, Minneapolis, MN, USA) in complete RPMI at 37 °C. Target tumor cell lines were labeled with lipophilic membrane dye DiO (5 µM, Invitrogen, Carlsbad, CA, USA) and 5 µM calcein red-orange (Invitrogen, Carlsbad, CA, USA) at 37 °C for 30 min. Target cells were washed three times with 1X PBS and were incubated with Cell Trace Violet labeled neutrophils in a round bottom plate at a 5:1 E:T ratio with and without human IgG1 antibodies for 4 h at 37 °C. Cells were then washed and labeled with Ghost Dye Red 780 (Tonbo Biosciences, San Diego, CA, USA) to measure viability. Cells were fixed with 0.5% (*w*/*v*) PFA, 1% (*w*/*v*) BSA, and 20 mM NaF (Sigma-Aldrich, St. Louis, MO, USA), washed with FACS buffer, and resuspended for analysis on an LSRFortessa (BD Biosciences, San Jose, CA, USA).

### 2.13. Complement-Dependent Cytotoxicity Assay

Experiments were performed by exposing cells in the presence of 10 µg/mL human IgGs to 15% human serum (GeminiBio, West Sacramento, CA, USA) in complete RPMI and staining them with the viability dye Ghost Dye Red 780 as previously described [16]. Cells were washed and resuspended for analysis on an LSRFortessa (BD Biosciences, San Jose, CA, USA).

### 2.14. Glycosylation Enzyme Inhibition

The sialic acid enzyme neuraminidase at 1:1000 dilution (Sigma Aldrich, St. Louis, MO, USA) and O-glycosylation inhibitor Benzyl 2-acetamido-2-deoxy-α-D-galactopyranoside hydrate (B2a2, Sigma Aldrich, St. Louis, MO, USA) at 2 mM were incubated with cells for 2 h or 40 h, respectively, before staining with anti-MUC1 antibodies.

### 2.15. Glycopeptide ELISA

Peptides and glycopeptides were synthesized as previously described [14,18,19]. Immulon 4HBX ELISA plates (Thermo Fisher Scientific, Waltham, MA, USA) were coated overnight with 10 µg/mL glycopeptides in 1X PBS in duplicate wells. Plates were blocked for 1 h at room temperature with 2.5% BSA. Primary anti-human MUC1 IgG antibodies were diluted to 5 µg/mL in 2.5% BSA and incubated for 1 h at RT. Plates were washed five times with 0.1% TWEEN20 wash solution, and then a 1:1000 dilution of goat anti-human IgG-HRP (SouthernBiotech, Birmingham, AL, USA) was used as a secondary, followed by five more washes before the addition of TMB substrate (BioLegend, San Diego, CA, USA). The reaction was quenched with 2N H_2_SO_4_ stop solution and read at 450 nm on a SpectraMaxi3 (Molecular Devices, San Jose, CA, USA).

### 2.16. MCP-1 ELISA

An MCP-1 matched antibody pair set (Sino Biological, Beijing, China) was used in a sandwich ELISA assay with Immulon 4HBX ELISA plates according to the manufacturer’s instructions. The range of its detection, ~4 pg/mL to 250 pg/mL, encompassed what was observed in the cytokine bead array assay. Briefly, capture antibody was diluted to 2 μg/mL in PBS and used to coat the plates. After the incubation of co-culture supernatants from the ADCR assay, rabbit anti-Human MCP-1/CCL2 conjugated to horseradish-peroxidase at 0.5 μg/mL was used to detect cytokine levels following the addition of TMB substrate. The reaction was quenched with 2N H_2_SO_4_ stop solution and read at 450 nm on a SpectraMaxi3 (Molecular Devices, San Jose, CA, USA).

### 2.17. Imaging Flow Cytometry

CellTrace Yellow-labeled Raji MUC1 22TR target cells were co-incubated 1:1 with CellTrace Violet-labeled THP-1 effector cells and human IgG1 antibodies in a phagocytosis assay for 1 h. Cells were then stained with Ghost Dye Red 780 dye (Tonbo Biosciences, San Diego, CA, USA) for 15 min. Half of each sample was run on a conventional flow cytometer (LSRFortessa, BD Biosciences, San Jose, CA, USA), and the other half was acquired on an AMNIS Image Stream Cytometer (Cytek Biosciences, Fremont, CA, USA). On the conventional cytometer, cells were gated to be the percentage of CellTrace-Yellow+ cells of CellTrace-Violet+ THP-1 cells. On the imaging cytometer, cells were first visualized by bright field and then identified as Raji MUC1 22TR targets (PE channel) or THP-1 monocytes (PacBlue channel). The IDEAS software v6.2 was used to evaluate the percentage of images that contained single live THP-1 cells with dual CellTrace-Yellow and CellTrace-Violet fluorescent signals that also received a positive internalization score on the PE-channel measured with the software’s built-in internalization wizard. Single-channel images were merged to confirm the uptake of Raji MUC1 22TR cells by THP-1 cells.

### 2.18. Data Analysis

Data visualization and statistical analyses were performed in FlowJo v10 and GraphPad Prism v10. Results were represented as mean ± standard error of the mean (SEM) as specified in each figure legend. Statistical tests for each dataset are listed in each figure legend. Significance for all experiments was defined as follows: * *p* < 0.05, ** *p* < 0.01, *** *p* < 0.001, **** *p* < 0.0001.

## 3. Results

### 3.1. Characterization of the Target Cell Lines and Antibody Binding

Due to polymorphisms in the MUC1 VNTR region that can lead to large differences in the number of epitopes in each allele, cell lines were selected that lacked surface MUC1 expression. These were transfected with defined MUC1 genes to be able to ask specific questions about the role of epitope abundance, proximity, and association with the membrane in various antibody-mediated functional assays. As one target, we chose the MUC1-negative Jurkat cell line that has a mutation in the COSMC (*C1GALT1C1*) gene, a common perturbation in many human tumors (Catalogue Of Somatic Mutations In Cancer Database v100, https://cancer.sanger.ac.uk/cosmic accessed on 21 September 2024). Without a functional COSMC chaperone, Jurkat cells have surface proteins with very short O-linked sugars. Transducing this cell line to express MUC1 containing 22 tandem repeats (Jurkat MUC1 22TR) mimics MUC1 hypoglycosylation commonly observed in tumors derived from patients. We also transduced Jurkat cells to express CD20 (Jurkat CD20), the target for the anti-CD20 antibody rituximab, a positive control in functional studies. As a second target, we transduced Raji cells with MUC1 22TR-generating Raji MUC1 22TR cells. Raji cells have intact *C1GALT1C1* expression, but nevertheless they express hypoglycosylated MUC1 [20,21]. Raji cells endogenously express CD20, and that afforded us the ability to use a cell line that co-expressed the two surface molecule targets. A comparison of total surface MUC1 and CD20 expression on the parental and other control cells together with the transduced cell lines is shown in Figure S2A.

We stained these new targets with 12 cloned human anti-MUC1 antibodies [14] and used trastuzumab (Herceptin, anti-HER2) and rituximab (Rituxan) as human IgG1 antibody controls (Figure S2B). Neither target expressed HER2 (*ERBB2*) [22], so Herceptin was used in these and other experiments as an IgG1 isotype control. H17K7 and H22K7, anti-MUC1 mAbs specific for a GSTAPP epitope, did not recognize MUC1 on either target cell line, consistent with the low binding observed previously on epithelial tumor lines [14]. The three antibodies displaying the highest binding on Jurkat MUC1 22TR cells were selected for further assays: H4K11 that binds “APPHGVTS” and H15K6 and H19K6 that recognize “PDTRP”.

**Figure 1:**
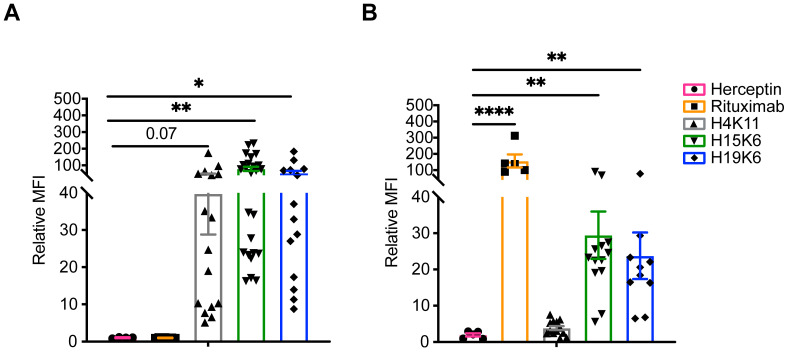
Binding of human anti-MUC1 mAbs and control antibodies on target cells. Surface staining with indicated antibodies and goat anti-human IgG secondaries on Jurkat MUC1 22TR (**A**) or Raji MUC1 22TR (**B**). As cytometer voltages were set independently for each experiment, the geometric mean fluorescence intensity (MFI) of each primary-antibody containing a sample was normalized to a secondary-only negative control. Each dot represents an individual staining (n = 4–25, Jurkat MUC1 22TR utilizing either PGK or EF1α promoters to drive *MUC1* gene expression; n = 5–13 Raji MUC1 22TR); bars depict mean ± SEM. Comparisons made by Kruskal-Wallis test with Dunn’s correction for multiple testing. * *p* < 0.05, ** *p* < 0.01, **** *p* < 0.0001.

More than a dozen staining experiments were performed, confirming high binding for all three mAbs on Jurkat MUC1 22TR cells (Figure 1A); however, much less binding of H4K11 was observed on Raji MUC1 22TR cells compared to H15K6 and H19K6 (Figure 1B). To better understand the specificity and tolerance of each of the antibodies for different sugar residues on the MUC1 peptide backbone, we performed an ELISA with multiple 20-mer glycopeptides (Figure S3A). As the H4K11 epitope spans the border of two tandem repeats, it only bound the 100-mer peptide that contained five contiguous 20-mer repeats. There were slight but significant changes in H15K6 and H19K6 binding when the serine or threonine proximal to the PDTRP sequence of the 20-mer was glycosylated with a Tn sugar. The binding of both antibodies was blocked with the triple-TF glycosylated and triple-Tn glycosylated peptides. Reduced but still present binding to TF-T9 and STn-T9 compared to Tn-T9 revealed greater tolerance for the shorter Tn sugar moiety. A ranking of H15K6 and H19K6 binding is shown in Figure S3B, revealing the importance for the T4 residue to be non-glycosylated, in addition to the T9 position.

### 3.2. Characterization of Anti-MUC1 Antibody-Mediated Effector Functions

#### 3.2.1. Cytokine Release

**Figure 2:**
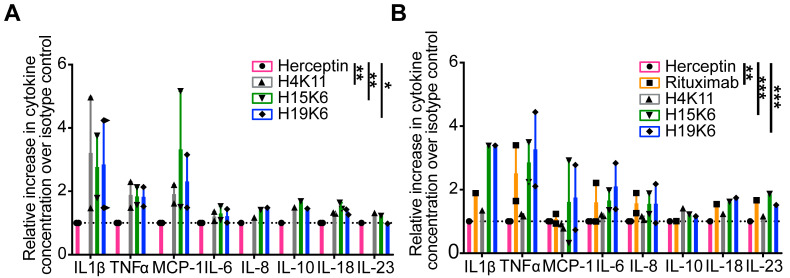
Human anti-MUC1 mAbs mediate antibody-dependent cytokine release (ADCR). Healthy donor peripheral blood mononuclear cells (PBMC) were incubated for four hours with the Jurkat MUC1 22TR (**A**) and Raji MUC1 22TR (**B**) cells at an effector to target ratio (E:T) of 25:1 and individual mAbs (10 µg/mL). Thirteen cytokines in the LEGENDplex Human Inflammation panel 1 were assayed from the supernatants of the co-incubations in two independent experiments. Cytokines measured above the limit of detection are shown. The gMFI of each cytokine was converted to absolute picogram quantities, and each was normalized to the isotype control Herceptin condition. All eight cytokines were simultaneously compared by antibody condition in a Kruskal-Wallis rank-sum test with Dunn’s correction for multiple comparisons; * *p* < 0.05, ** *p* < 0.01, *** *p* < 0.001.

We co-cultured the target cell lines with healthy donor peripheral blood mononuclear cells (PBMC) at a 25:1 E:T ratio and the three antibodies for four hours. The co-culture supernatants were collected, and IL-1β, IFN-α2, IFN-γ, TNF-α, MCP-1, IL-6, CXCL8 (IL-8), IL-10, IL-12p70, IL-17A, IL-18, IL-23, and IL-33 were quantified by bead array. IL-1β, TNF-α, MCP-1, IL-6, CXCL8 (IL-8), IL-10, IL-18, and IL-23 were detected above background (Figure S4A). Fold change above isotype control was compared for two independent experiments, as plotted in Figure 2. MCP-1 levels were additionally verified by ELISA (Figure S4B). Although few cytokines were significant after correction for multiple testing, examining all eight cytokines simultaneously in a rank-sum test revealed significantly more cytokines in cultures with Jurkat MUC1 22TR and H4K11 (adjusted *p* = 0.0072), H15K6 (adjusted *p* = 0.0013), and H19K16 (adjusted *p* = 0.0112), and in cultures of Raji MUC1 22TR with rituximab (adjusted *p* = 0.0051), H15K6 (adjusted *p* = 0.0002), and H19K6 (adjusted *p* = 0.0002), but not with H4K11 (adjusted *p* = 0.5315), consistent with reduced H4K11 binding on Raji MUC1 22TR cells.

#### 3.2.2. Antibody-Dependent Cytotoxicity (ADCC)

**Figure 3:**
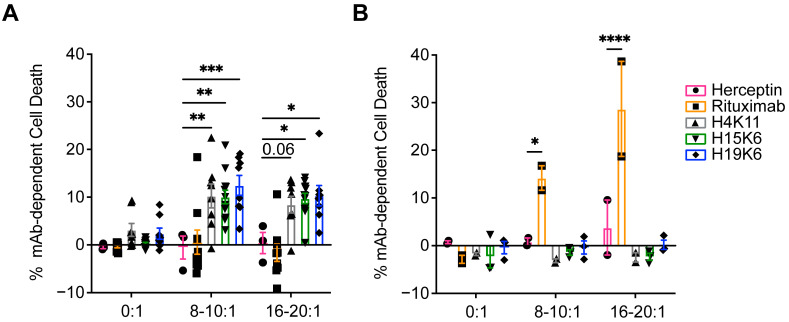
Human anti-MUC1 mAbs mediate antibody-dependent cellular cytotoxicity (ADCC). Jurkat MUC1 22TR (**A**) and Raji MUC1 22TR (**B**) targets were incubated with and without human NK cells from a healthy donor stimulated overnight with IL-2 at E:T ratios from 8:1 to 20:1 and the indicated human IgG antibodies. Each percentage of mAb-dependent cell death was calculated by taking the percentage of non-viable cells and subtracting non-antibody-mediated cell death that occurred in control wells without antibodies. Each dot plotted is the average of duplicates from n = 3–12 Jurkat MUC1 22TR or n = 2–3 Raji MUC1 22TR independent experiments. Bars depict the mean ± SEM. Data were compared by two-way ANOVA with Šídák’s multiple comparisons test; * *p* < 0.05, ** *p* < 0.01, *** *p* < 0.001, **** *p* < 0.0001.

To test ADCC function, Jurkat MUC1 22TR and Raji MUC1 22TR cells were co-cultured with the antibodies and various ratios of magnetically enriched human NK cells. After a 4 h incubation, Jurkat MUC1 22TR cell viability was measured by flow cytometry and found to be reduced in wells with H4K11, H15K6, and H19K6, whereas only rituximab mediated the ADCC of Raji MUC1 22TR cells (Figure 3). Similar incubation of tumor cells and antibodies alone did not result in any direct effect of antibodies on cell viability (Figure 3).

#### 3.2.3. Antibody-Dependent Phagocytosis (ADCP)

**Figure 4:**
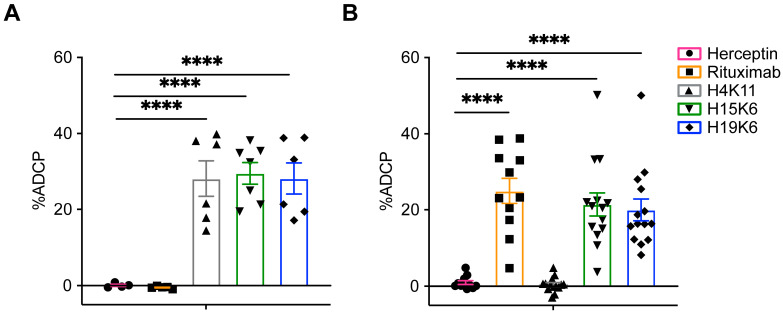
The ability of human anti-MUC1 mAbs to mediate antibody-dependent cellular phagocytosis (ADCP) is target-dependent. CellTrace Yellow-labeled Jurkat MUC1 22TR (**A**) and Raji MUC1 22TR (**B**) target cells were coincubated 1:1 with CellTrace Violet-labeled human THP-1 monocytes. %ADCP was calculated by taking the percentage of THP-1 cells that were also CellTrace Yellow+ and subtracting the background of double positive events that occurred with no primary mAbs. Plotted for each dot is the average of duplicates from n = 4–7 Jurkat MUC1 22TR or n = 11–14 Raji MUC1 22TR independent experiments. Bars depict the mean ± SEM. Statistical comparisons were made through one-way ANOVA with Dunnett’s test to correct for multiple comparisons, **** *p* < 0.0001.

Phagocytosis by monocytes and macrophages is another method of tumor cell elimination that is aided by direct tumor-targeting antibodies. Jurkat MUC1 22TR and Raji MUC1 22TR cells were incubated with the antibodies and the monocytic THP-1 cell line in a 1:1 ratio. All three MUC1 antibodies facilitated ADCP of Jurkat MUC1 22TR cells (Figure 4A), whereas only rituximab, H15K6, and H19K6 mediated ADCP on Raji MUC1 22TR cells (Figure 4B), again consistent with the ADCR assay (Figure 2) and antibody binding results (Figure 1 and Figure S5).

#### 3.2.4. Antibody-Dependent Trogocytosis/Trogoptosis (ADCT)

**Figure 5:**
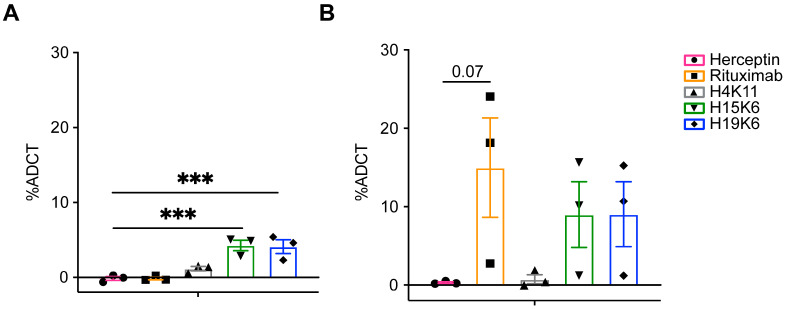
Human anti-MUC1 mAbs mediate antibody-dependent cellular trogocytosis. Jurkat MUC1 22TR^hi^ (**A**) and Raji MUC1 22TR (**B**) target cells labeled with calcein-red and DiO and coincubated with CellTrace Violet-labeled healthy donor neutrophils stimulated overnight with IFNγ and G-CSF, at an E:T ratio of 5:1 for 4 h with and without antibodies. Antibody-dependent cellular trogocytosis was measured by taking the %DiO+Calcein−CellTrace+ neutrophils and subtracting the same percentage in wells with no primary antibodies, representing the background trogocytosis. Each dot shows the average of duplicates from n = 3 independent experiments. Bars depict the mean ± SEM. Statistical comparisons were made through one-way ANOVA with Dunnett’s test to correct for multiple comparisons; *** *p* < 0.001.

Another recently described mechanism of antibody-mediated tumor cell killing is trogoptosis by neutrophils that trogocytose portions of tumor cell membranes [17]. In each assay, the membrane dye DiO labeled target cell membranes, calcein-red labeled each target cell’s cytoplasm, and CellTrace Violet labeled neutrophils. We observed increased trogocytosis of Jurkat MUC1 22TR and Raji MUC1 22TR target cell membranes with H15K6 and H19K6, as well as rituximab on Raji MUC1 22TR cells (Figure 5 and Figure S6). This occurred without any significant target cell death (Figure S7A). We observed instead a loss in calcein intensity in the target cells in the co-cultures containing H15K6 and H19K6, compared to the isotype control (Figure S7B).

#### 3.2.5. Antibody-Dependent Complement-Dependent Cytotoxicity (ADCDC)

**Figure 6:**
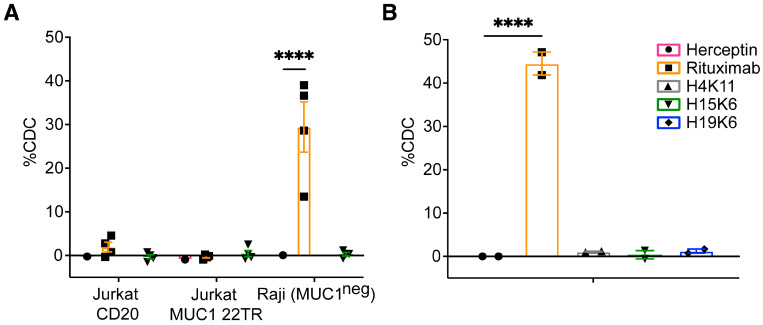
Human anti-MUC1 mAbs do not mediate complement-dependent cytotoxicity (CDC). Jurkat MUC1 22TR (**A**) and Raji MUC1 22TR (**B**) target cells were incubated for 30–120 min with human mAbs (10 µg/mL) and 15% normal human serum. Jurkat CD20 and parental Raji cells were used as additional controls (**A**). %CDC was calculated by taking the percentage of non-viable cells and subtracting the background cell death that occurred with no primary mAbs. Each dot shows a single value or average of duplicates from n = 2–4 independent experiments. Bars depict the mean ± SEM. Statistical comparisons were made through two-way (**A**) and one-way (**B**) ANOVA with Dunnett’s test to correct for multiple comparisons; **** *p* < 0.0001.

Finally, we examined whether ADCDC occurred in the presence of the anti-MUC1 antibodies and human serum. There was no CDC activity either on the Jurkat MUC1 22TR line in the presence of anti-MUC1 antibodies or on Jurkat CD20 cells in the presence of rituximab (Figure 6). Raji MUC1 22TR cells were susceptible to CDC in the presence of rituximab, but no CDC activity was observed with H4K11, H15K6, or H19K6 (Figure 6).

### 3.3. Characterization of MUC1 Antigen Attributes That Impact mAb-Epitope Interactions and Effector Functions

**Figure 7:**
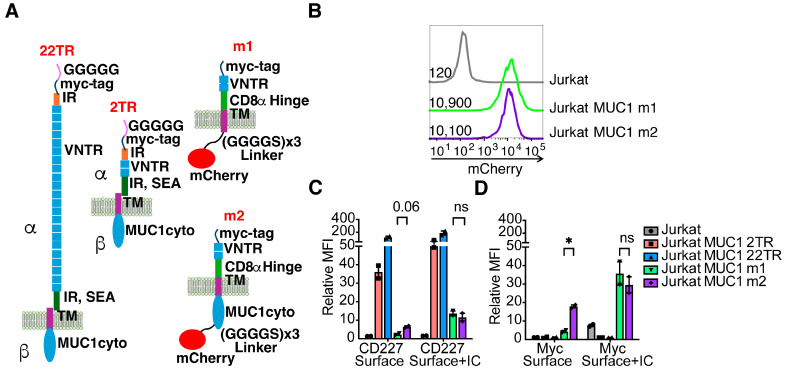
MUC1 constructs that vary in epitope number, proximity to membrane, and retention on cell surface. (**A**). 22TR: MUC1 with 22 tandem repeats of 20 amino acids from the VNTR region. It can be cleaved in the SEA domain into alpha and beta subunits, which remain non-covalently associated. 2TR: MUC1 construct with 2 tandem repeats of 20 amino acids from the VNTR region. m1: MUC1 with 2 tandem repeats, no cleavable domain or intracellular cytoplasmic domain, and an mCherry reporter and an N-terminal myc tag. m2: MUC1 with two tandem repeats, no cleavable domain, the MUC1 cytoplasmic domain, and an mCherry reporter and an N-terminal myc tag. N = amino-terminus, C = Carboxy-terminus, IR = imperfect repeats, VNTR = variable number tandem repeats, SEA = sperm protein, enterokinase, agrin domain TM = transmembrane. (**B**). The level of mCherry expression representing overall MUC1 variant expression of m1 and m2 in lentivirally transduced Jurkat cells as measured by flow cytometry. Numbers represent the geometric MFI for each population of cells. Parental Jurkats are untransduced and shown as a negative control. (**C**,**D**). Geometric mean fluorescent intensity (gMFI) of anti-CD227 (**C**) or anti-myc antibody staining (**D**) on the surface of intact cells (Surface) or combined surface and intracellular staining (Surface + IC). Relative MFI is the gMFI normalized to the gMFI background on unstained cells. Each dot represents one of two independent experiments. Each bar is the mean, and error bars depict ± SEM. The relative antibody binding of CD227 and myc-tag between m1 and m2 cells was compared by student’s *t*-tests with Holm-Sidak’s correction method for multiple comparisons, * *p* < 0.05, ns = not significant.

With a better understanding of which antibody-dependent functions the human anti-MUC1 antibodies could mediate, we set out to investigate specific MUC1 antibody-epitope interactions and how they affect each antibody’s efficacy. We generated four additional Jurkat MUC1 cell lines with different MUC1 constructs (Figure 7). To examine the influence of epitope distance from the target cell surface, we transduced Jurkat cells with MUC1 containing only two tandem repeats, MUC1 2TR, to compare with Jurkat MUC1 22TR. MUC1’s extracellular domain is naturally associated with the membrane through an SEA domain, leaving it susceptible to dissociation from the cell surface [23,24]. We designed MUC1 constructs, termed “m1” and “m2”, modifying the extracellular domain to remain permanently associated with the membrane by replacing the imperfect repeat (IR) and SEA domains with a recombinant CD8α-hinge transmembrane domain that is commonly used in CAR-T cell engineering (Supplemental Materials) and transduced Jurkat cells with these constructs. The m1 construct additionally lacks the MUC1 cytoplasmic domain, which is intact in m2. Modifying the intracellular signaling domain is expected to prevent MUC1 recycling through endosomes [25]. Included in the m1 and m2 constructs was a fusion via a glycine serine linker to mCherry to allow us to monitor total construct expression. We also added a myc-tag at the N-terminus of all four constructs to enable us to quantify the total surface expression of each molecule without the confounding factor of having variable numbers of epitopes and any changes in glycosylation that could affect the ability of anti-MUC1 antibodies to bind (Figure S8). Gating the cells into bins with defined average copies of MUC1 on their surface using the myc-tag antibodies (Figure S8A) revealed more efficient binding of H15K6 to the m1 and m2 than to MUC1 2TR, whereas the opposite was true for the mouse anti-MUC1 antibody HMPV that bound more to MUC1 2TR than to m1 or m2 (Figure S8B–E). This suggests that m1/m2 and MUC1 2TR constructs could be differentially glycosylated. As expected, the most MUC1 antibody binding per molecule was observed with the MUC1 22TR due to the larger number of epitopes present in each molecule and the ability for more than two antibodies to bind each protein (Figure S8).

Combined analysis of anti-myc, anti-MUC1, and mCherry expression revealed that the intracellular signaling domain influences the retention and/or recycling of the MUC1 molecule up to the surface, as both Jurkat MUC1 m1 and Jurkat MUC1 m2 cell lines had similar levels of mCherry (total expression) (Figure 7B), whereas the Jurkat MUC1 m1 line had much reduced surface anti-myc and anti-MUC1 binding (Figure 7C). Staining permeabilized cells to capture both surface and intracellular MUC1 revealed comparable overall expression, correlating well with the mCherry results (Figure 7C). While the MUC1 intracellular domain has been described to influence glycosylation patterns [26], we did not observe any differences in H15K6 binding (Figure S8E). Therefore, changes in sugar residues that would impact the H15K6 epitope do not appear to be different.

**Figure 8:**
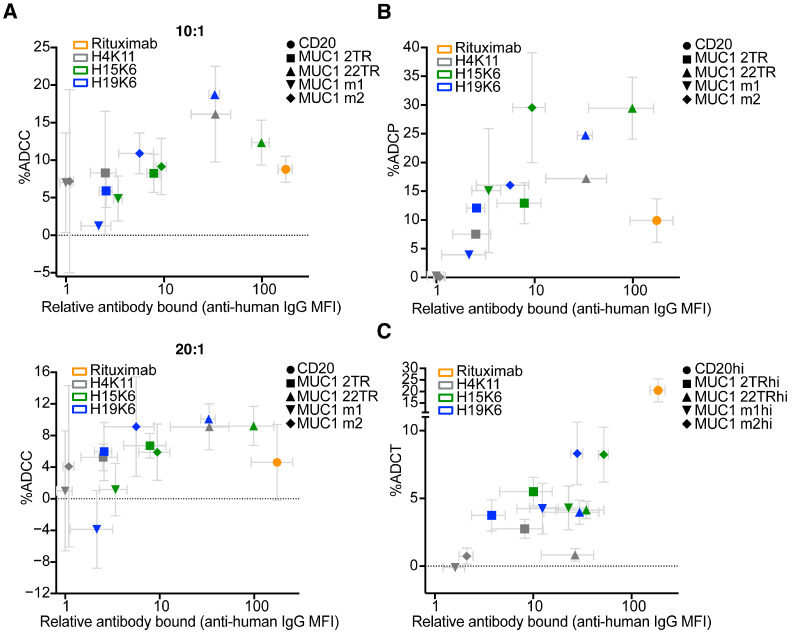
Amount of antibody bound does not correlate with efficiency of ADCC or ADCP but does with ADCT. Cells were stained with 10 µg/mL of each primary antibody for 15 min prior to (**A**) 4 h incubation with human NK cells that had been stimulated overnight with IL-2 at effector to target ratios ranging from 10:1 to 20:1, (**B**) 1 h incubation with human THP-1 cells at a 1:1 ratio, or (**C**) 4 h incubation with human neutrophils stimulated overnight with hIFNγ and G-CSF. Jurkat cells transduced with human CD20 were used with rituximab as positive controls. %ADCC, ADCP, and trogocytosis were calculated as in Figure 3, Figure 4 and Figure 5. Gray error bars represent mean ± SEM for average relative MFI and % antibody-mediated function. There is no significant correlation between the amount of antibody bound (average relative MFI) and average %ADCC (r^2^ = 0.07, ns 10:1; r^2^ = 0.05, ns 20:1) or %ADCP (r^2^ = 0.06, ns), but there is for trogocytosis (r^2^ = 0.81, *p* < 0.0001).

Using Jurkat CD20, 2TR, 22TR, m1, and m2 along with antibodies H4K11, H15K6, and H19K6 and human NK cells at 20:1 and 10:1 effector to target ratios, we measured the ability of each antibody to mediate ADCC relative to its surface staining level. At both effector-to-target ratios, NK cells killed Jurkat MUC1 22TR cells bound by anti-MUC1 antibodies more efficiently than Jurkat CD20 cells bound by much more rituximab (Figure 8A). Considering all cell line-antibody combinations, there was no significant correlation between the amount of antibody bound to the target cell and ADCC (Pearson r = 0.27,10:1; r = 0.23, 20:1). The efficiency of ADCP with H15K6 bound to m2 cells was equal to H15K6 bound to cells carrying 22TR, despite almost 10X more antibody being bound to the 22TR targets (Figure 8B). There was also considerably more ADCP of Jurkat MUC1 m2 cells with H15K6 than of Jurkat MUC1 2TR cells with a similar amount of H15K6 bound (Figure 8B). Similar to ADCC, when taking into account all cell line-antibody combinations, there was no significant correlation of antibody bound with ADCP (Pearson r = 0.24). In part driven by efficient trogocytosis of CD20 bound by rituximab, there was a positive correlation between the amount of antibody bound and ADCT (Pearson r = 0.9265; *p* < 0.0001), as shown in Figure 8C.

Examination of MUC1-expressing cell lines alone without the inclusion of the Jurkat CD20-rituximab data also showed no significant correlations for ADCC (Pearson r = 0.545, ns, 10:1; r = 0.52, ns, 20:1). There was then a mild but significant correlation of %ADCP with the amount of antibody bound (Pearson r = 0.65, *p* = 0.02), and ADCT showed no significant correlation (Pearson r = 0.56, ns).

To further assess the role of the amount of antibody-bound, transduced cells in each line that bound high (hi), intermediate (int), and low (lo) amounts of the anti-myc antibody were separately sorted. Lentiviral transduction resulted in stable expression, so each separate hi, int, and lo cell line maintained distinct distributions of antibody binding as cell lines were cultured over several passages. Each of these sorted cell lines was used in the ADCC, ADCP, and ADCT assays, and the average amounts of antibodies bound were plotted versus the % effector function observed. Similar results were observed with the five parental lines alone, in that only %ADCT was correlated with more bound antibody (Figure S9). Table S2 summarizes the correlations between the amount of antibody bound and effector functions across each MUC1 construct individually, combining data points from all sorted hi, int, and lo cell lines for each MUC1 variant. There is a strong trend for significant correlations of functional efficiency within each MUC1 variant class. Therefore, the lack of correlations in all variants combined supports that epitope properties such as proximity to and permanent association with the cell membrane drive differences between cell lines.

## 4. Discussion

While monoclonal antibodies targeting checkpoint blockade molecules operate by broadly activating the immune system, monoclonal antibodies are directed to specific tumor antigens and affect their functions specifically at the tumor site. They can block signaling, act as Trojan horses carrying toxic compounds, coordinate innate immune engagement for cytotoxicity through cellular and non-cellular means, and promote antigen uptake to jumpstart adaptive immunity that can provide durable protection [27]. Despite their great potential and resources put into their development, very few monoclonal antibodies have some or most of these properties and have successfully reached clinical practice. The 43 antibodies approved to treat cancer (as of June 2024) are directed against 22 molecular targets [28]. Furthermore, many of these antibodies were generated initially in mice, so despite adding human Fc domains and humanization, these antibodies can still be immunogenic, leading to reductions in therapeutic efficacy due to immune reactivity against them [29]. A major problem also facing the field is how to efficiently and effectively test anti-cancer antibodies in ways that are predictive of responses in humans before costly clinical trials are initiated [30]. Lastly, the rules governing what makes a good antigen target and the mechanisms by which these monoclonal antibodies exert their anti-tumor function are still poorly understood.

We set out to address whether three fully human anti-MUC1 antibodies, H4K11, H15K6, and H19K6, possess properties that would make them candidates to add to the small but growing list of effective direct-targeting monoclonal antibody therapeutics. Each of the monoclonals isolated from a vaccinated individual was indeed found to facilitate numerous immune effector functions. All three mAbs were shown to be capable of inducing ADCR, ADCC, and ADCP, while H15K6 and H19K6 could additionally mediate ADCT, and none of the three mAbs tested facilitated CDC with human serum. Throughout the course of characterization of these immune effector functions, it became clear that the circumstances under which each of these monoclonals can be most effective vary. The use of multiple cell lines and MUC1 antigen variants helped to tease out different factors that most strongly influence antibody efficacy in eliminating target tumor cells.

In addition to antibody affinity/avidity impacting the amount of bound therapeutic antibody to a target cell, differences in surface antigen expression levels can also dictate the amount of antibody bound and degree of therapeutic mAb efficacy. Indeed, high antigen expression and antibody binding have been shown to correlate with increases in some but not all effector functions [31,32,33,34,35]. The lack of ADCC observed with H15K6 and H19K6 on Raji MUC1 22TR cells may be due to the much lower expression of MUC1 than CD20 on these cells that still show ADCC activity with rituximab. As shown in Figure 1B, rituximab bound Raji MUC1 22TR cells approximately five times more than H15K6 or H19K6. While overall MUC1 22TR expression does not seem to be much lower in Raji MUC1 22TR cells compared with Jurkat MUC1 22TR cells, Raji cells have been shown to be sensitive to antigen abundance/antibody binding levels for effective ADCC, and the Raji cell line has been referred to in the literature as “NK-resistant” [36,37,38,39]. In addition to the target cell upregulation of MHC molecules, ADCC can be inhibited through target cell sialic acids binding to siglec molecules on NK cells [39,40,41]. Treatment of Raji MUC1 22TR cells with neuraminidase revealed more anti-hypoglycosylated MUC1 antibody epitopes (see Figure S10C), providing evidence of cell-surface sialic acids present in these cells. Taken together, these results could help explain why the threshold to achieve ADCC in Raji MUC1 22TR cells is higher and can only be surmounted by rituximab binding at a level five times greater than any of the MUC1 antibodies.

Two recent trogoptosis studies have described how certain cells can be resistant to cell death from neutrophils while they still remain susceptible to trogocytosis in the presence of target antibodies [42,43]. In ADCT assays using anti-MUC1 mAbs and activated neutrophils, we similarly detected susceptibility to trogocytosis, indicated by a reduction in the calcein red signal labeling intracellular proteins. However, there was no corresponding increase in the Ghost Dye Red 780 viability stain, implying no heightened cell death or trogoptosis in this setting.

For some anti-tumor antibody targets, the distance of the epitope from the cell membrane plays a crucial role in effector function efficiency, though the perceived preference of each mechanism for distal vs. proximal epitope location varies. ADCC and CDC are more efficient when antibodies target cell membrane-proximal epitopes, while this has not always held for ADCP [32,44]. Most FDA-approved antibodies target epitopes within <10 nm of the cell surface [44]. ADCC in particular is thought to require a close synapse between NK cells and target cells for the release and uptake of cytotoxic granules [45]. Despite its large size, MUC1 is flexible and folds back on itself and can therefore simultaneously present epitopes both proximal and distal to the cell surface [46]. Our results here confirm proximal, membrane-anchored epitopes as optimal targets. However, it is important to note that while the m2 MUC1-transfected cells were often more efficiently eliminated compared to the 22TR MUC1 cells, ADCC and ADCP functions on cells with the MUC1 22TR antigen were superior to ADCC and ADCP on CD20 targets with much higher levels of rituximab bound (Figure 8).

In addition to the epitope distance from the membrane, the intra-membrane distribution of the target and its ability to cluster have been shown to be important for efficient initiation of the complement cascade and for FcR-clustering on effector cells [37,47,48,49]. MUC1 has already been shown to segregate into lipid rafts [50,51]. That and the VNTR region to which multiple antibodies can bind is likely to better promote complement factor and/or FcR clustering than a single epitope on a given antigen.

Recent CRISPR screens to identify features that lead to resistance to ADCC and ADCP have had mucins and several molecules in the O-glycosylation pathway, including COSMC and T-synthase, emerge as significant hits [52,53,54]. In other studies, the expression and cell-surface co-localization of mucins with targets such as HER2 or EGFR were shown to cause resistance to ADCC [55,56,57]. Despite MUC1’s ability to inhibit responses to other monoclonal therapies, we show here that targeting MUC1 directly is still a promising therapeutic approach that may also be useful in combination with other therapies that it currently appears to inhibit.

This study contributes to the growing understanding of how certain cell lines/tumor types can be more or less susceptible to a particular mechanism of antibody-mediated immune effector function, which highlights the need for and importance of identifying pathways that could be targeted in combination with tumor-targeting antibodies, similar to how immune checkpoint therapies have come to be used in combination with one another and with other emerging therapeutic strategies. The observation of varying tumor cell susceptibility to immune-mediated killing extends not only to direct-tumor targeting antibodies described here and elsewhere [58,59,60] but to all cellular mechanisms of tumor cell elimination, including those that involve anti-tumor T cells bearing tumor-specific TCRs or CARs [61]. Future studies should explore whether combining anti-MUC1 antibodies with CD24 or CD47 blocking mAbs, for instance, which inhibit the “don’t eat me” signal on tumor cells, could further increase ADCP and ADCT as has been shown for HER2- and CD20-expressing cells upon administration of their respective targeting antibodies [17,62,63]. Combination therapies with agonistic anti-CD27 agents or pegfilgrastim may also promote better myeloid cell recruitment and anti-tumor activity [64,65]. Strategies to couple enzymes to antibodies to make local changes to cell surface sialylation, such as the ones Bertozzi and colleagues have begun testing, may also be a valuable combination strategy for future investigations [41].

While the explorations of effector functions in this manuscript were limited to cells of hematopoietic origin, the additional distribution of overexpressed MUC1 across various solid tumor types accounts for its presence in up to 80% of human cancers [66,67] and continues to make MUC1 an attractive target for immunotherapy. Future work to investigate in depth whether human anti-MUC1 mAbs can mediate any additional antibody effector mechanisms relevant for non-hematopoietic cells is of great interest. In addition, it will be necessary to more fully understand and verify the current findings to bolster consideration of these mAbs as potential solid tumor therapeutics. In Lohmueller et al., some antibody effector functions for human MUC1 mAbs were described on cells of epithelial origin, including CDC on ZR-75-1 breast cancer cells [14]. In our investigations, we did not find H15K6 capable of mediating CDC on Jurkat MUC1 22TR cells, likely due to the co-expression of complement inhibitory factors such as CD59 [68,69], as Jurkat CD20 cells were also not lysed by rituximab. There was also a lack of CDC with H15K6 and Raji MUC1 22TR cells, a cell line otherwise quite susceptible to CDC with the control IgG rituximab. The discrepancy in CDC capacity between Raji and ZR-75-1 experiments could potentially be explained by different levels of sialylation across the cell lines [70] or by the different sources of complement used in each assay. Here, normal human serum was selected, which most closely mimics what would be seen in vivo, whereas with ZR-75-1 cells, baby rabbit complement was used [14]. Baby rabbit complement can bypass some complement inhibitory factors and can overestimate responses [71]. CDC has also been shown to be influenced by epitope distance from the membrane [32,72,73], with more proximal epitopes showing greater efficiency [73,74]. ZR-75-1 cells were not transduced and expressed MUC1 endogenously. It is unknown if they express any of the splice variants of MUC1 that would result in shorter antigens/more proximal epitopes [75]. Or, alternatively, if the epitopes remain quite distal from the membrane, it is possible baby rabbit complement has a longer half-life, increasing the likelihood of the C3 complex to form, even if further from the membrane [32]. We had hoped to test Raji MUC1 2TR cells for susceptibility to CDC with a more proximal epitope for anti-MUC1 mAbs; however, they did not sufficiently underglycosylate the MUC1 molecule, and the antibodies recognizing hypoglycosylated MUC1 were unable to bind (Figure S10).

Although we only used a single concentration of antibody for all functional assays sufficient to detect all effector mechanisms measured, more subtle differences in antibody efficacy and mechanisms of action could be discovered across a wider dose range. More detailed physiochemical characterization of these mAbs could also reveal additional attributes that contribute to their function and remains an area of interest for future investigation.

Our studies utilized mAbs with an unmodified IgG1 Fc domain. Gong et al. recently tested a defucosylated version of a humanized murine anti-Tn MUC1 clone, 5E5, and showed that it had enhanced ADCC [76]. In addition to fucose removal, many different modifications from the switched isotype class, altered glycosylation, and mutations that impact the binding affinity of the Fc domain have been discovered that can enhance mAb effector functions [77] and the incorporation of one or more of those with the anti-MUC1 Fab domains described here may further enhance the efficacy of these anti-MUC1 antibodies.

In conclusion, at least three fully human anti-MUC1 antibodies that were identified through prophylactic vaccination can facilitate ADCC, ADCR, ADCP, and ADCT on tumor targets. These results suggest antibodies resulting from vaccination could lead to anti-tumor functions within vaccinated individuals. Unique structural properties inherent to the MUC1 antigen afforded the ability to examine epitope features that contribute to the antibody-epitope interaction and influence the degree of immune effector functions. These data provide further evidence that other factors aside from antigen abundance can drive strong immune responses with direct-targeting anti-tumor monoclonal antibodies. These properties and the inclusion of multiple cell lines/tumor types should be considered in the design and future development of therapeutics for MUC1 and other key tumor targets.

## Data Availability

The data presented in this study are openly available in Pennsieve. https://doi.org/10.26275/pgzg-jlcs (accessed on 21 September 2024).

## Supplementary Materials

The following supporting information can be downloaded at: https://www.mdpi.com/article/10.3390/antib13040085/s1, Figure S1: Flow chart of functional assay procedures; Figure S2: Binding of the human anti-MUC1 mAbs and control antibodies on different target cells; Figure S3: Glycosylation of select serine and threonine residues within the MUC1 tandem repeat can alter anti-human MUC1 IgG binding; Figure S4: Antibody-mediated cytokine release by PBMCs co-cultured with Raji MUC1 22TR and Jurkat MUC1 22TR in the presence of indicated antibodies; Figure S5: Imaging cytometry confirms human anti-MUC1 mAbs mediate ADCP; Figure S6: Gating strategy for ADCT experiments; Figure S7: Measurements of cell death during antibody-dependent cellular trogocytosis assays; Figure S8: Dual staining with anti-myc and anti-MUC1 antibodies estimates the number of IgGs bound per MUC1 variant molecule; Figure S9: Amount of antibody bound correlates with efficiency of ADCC and ADCP within but not across cell lines possessing various levels of MUC1 variants; Figure S10: Human anti-MUC1 mAbs do not efficiently stain Raji cells expressing shorter MUC1 variants with 2 tandem repeats; Table S1: DNA constructs used in the study [78,79,80,81,82,83,84]; Table S2: Summary of Pearson R correlation coefficients across all human IgG antibodies tested by parental cell line in each assay in Figure S9; Additional Supplemental Material: MUC1 m1 and m2 sequences.

## Abbreviations

ADCC = antibody-dependent cellular cytotoxicity; ADCP = antibody-dependent cellular phagocytosis; ADCT = antibody-dependent cellular trogocytosis; CDC = complement dependent cytotoxicity; E:T = effector to target ratio; MUC1 = Mucin-1; NK = Natural Killer; PBMC = peripheral blood mononuclear cells; TR = tandem repeats.
