# Supplementary material for: Preventative Cancer Vaccine-Elicited Human Anti-MUC1 Antibodies Have Multiple Effector Functions
Source: Antibodies (Basel). 2024 Oct 10;13(4):85. doi: 10.3390/antib13040085 (PMC11503386; doi:10.3390/antib13040085)
Supplement: Supplementary file 1 [file antibodies-13-00085-s001.zip › antibodies-3227747-supplementary.pdf]

McKeague *et al.* Supplementary Material

Supplementary Tables S1-S2.
Supplementary Figures S1-S10.
Additional Supplemental Material
Supplemental References

Construct Insert Name	Backbone/Promoter	N-terminal tags	Reporter Gene(s)
2TR (*MUC1*)[1]	pSICO_EF1α[5]		
22TR (*MUC1*)[1]	pSICO_EF1α[5]		
2TR (*MUC1*)[1]	pHR_PGK[6]	polyglycine; myc-tag[7]	
22TR (*MUC1*)[1]	pHR_PGK[6]	polyglycine; myc-tag[7]	
m1 (*MUC1*)	pHR_PGK[6]	myc-tag[7]	mCherry[8]
m2 (*MUC1*)	pHR_PGK[6]	myc-tag[7]	mCherry[8]
CD20 (*MS4A1*)[2]	pHR_PGK[6]		
CD19 (*CD19*)[3]	pHR_PGK[6]		
Luc-ZsGreen[4]	pHIV_ZsGreen_EF1α[4]		Luciferase; ZsGreen[9]

Table S1. DNA constructs used in the study. 1. Sequence isolated from pcDNA3 constructs as described in Cascio et al., 2011 [1]. 2. Coding region from UniProt:P11836 3. Coding region from UniProt: P15391, same lentivirus described in Lohmueller et al. 2017 [2]. 4. pHIV-Luc-ZsGreen was a gift from Bryan Welm, (Addgene plasmid #39196; http://n2t.net/addgene:39196 ; RRID:Addgene_39196). 5. pSICO_EF1α came from the pHIV-Luciferase vector that was a gift from Bryan Welm (Addgene plasmid # 21375; http://n2t.net/addgene:21375 ; RRID:Addgene_21375) 6. pHR_PGK was a gift from Wendell Lim (Addgene plasmid # 79120; http://n2t.net/addgene:79120 ; RRID:Addgene_79120), Morsut et al., 2016 [3]. 7. Myc-tag, EQKLISEEDL, first described in Evan et al., 1985 [4], polyglycine LIPSTIC tag, GGGGG, an acceptor sequence for an attenuated Sortase A enzyme as described in Pasqual et al., 2018 [5]. 8. mCherry sequence first described in Shaner et al., 2004 [6]. 9. Luciferase is from Luc2P (firefly luciferase); ZsGreen first described in Matz et al., 1999 [7].

	MUC1 2TR	MUC1 22TR	MUC1 m1	MUC1 m2	CD20
16:1 ADCC	0.127	0.2817	** 0.7813	* 0.6176	# 0.7597
8:1 ADCC	* 0.5894	# 0.5172	** 0.7656	*** 0.878	# 0.8042
ADCP	*** 0.8794	** 0.7565	**** 0.9917	**** 0.943	** 0.9649
ADCT	*** 0.827	* 0.7033	*** 0.8299	*** 0.8703	**** 0.996

Table S2. Summary of Pearson R correlation coefficients across all human IgG antibodies tested by parental cell line in each assay in Figure S9. Means from n=3 replicate experiments were used to generate relative MFI by effector function paired values per parental cell line. Cells expressing MUC1 constructs had n=12 paired values (4 antibodies x 3 derived cell lines: hi, int, lo), while those expressing CD20 had n=6 paired values from the two antibodies tested with the three respective derived cell lines. #p<0.1, *p<0.05, **p<0.01, ***p<0.001, ****p<0.0001.

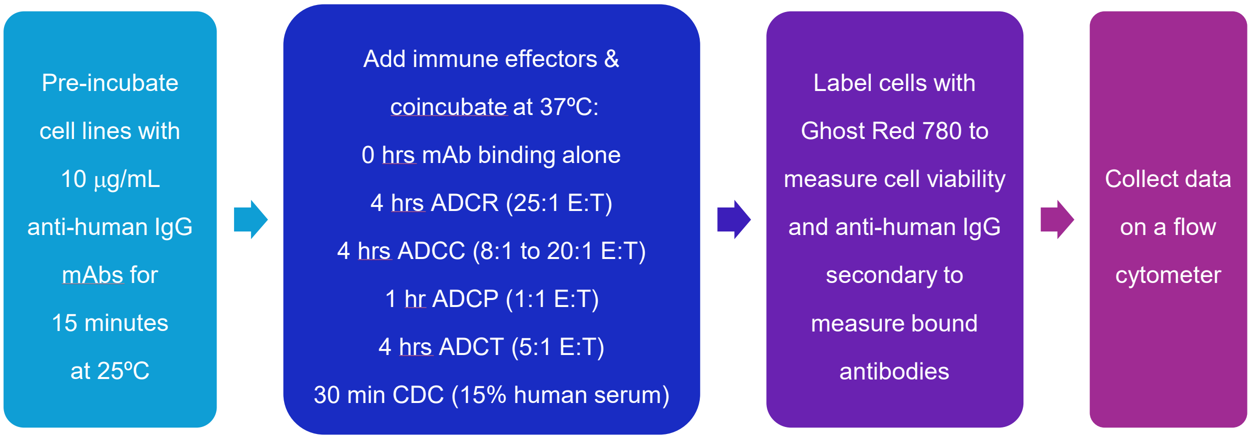

Figure S1. Flow chart of functional assay procedures. Lentivirally transduced Jurkat and Raji cell lines were pre-incubated with anti-human IgG1 mAbs for 15 minutes at room temperature prior to addition of immune effector cells: antibody-dependent cytokine release (ADCR): peripheral blood mononuclear cells (PBMC), antibody-dependent cellular cytotoxicity (ADCC): natural killer (NK) cells, antibody-dependent cellular phagocytosis (ADCP): THP-1 monocytes, antibody-dependent cellular trogocytosis (ADCT): neutrophils, or 15% normal human serum for complement-dependent cytotoxicity (CDC) assays. Fluorochrome-conjugated anti-human IgG secondary antibodies were added to measure levels of bound antibody on live cells either at the beginning (binding alone) or end of each respective assay. All functional assay readouts were collected on a flow cytometer.

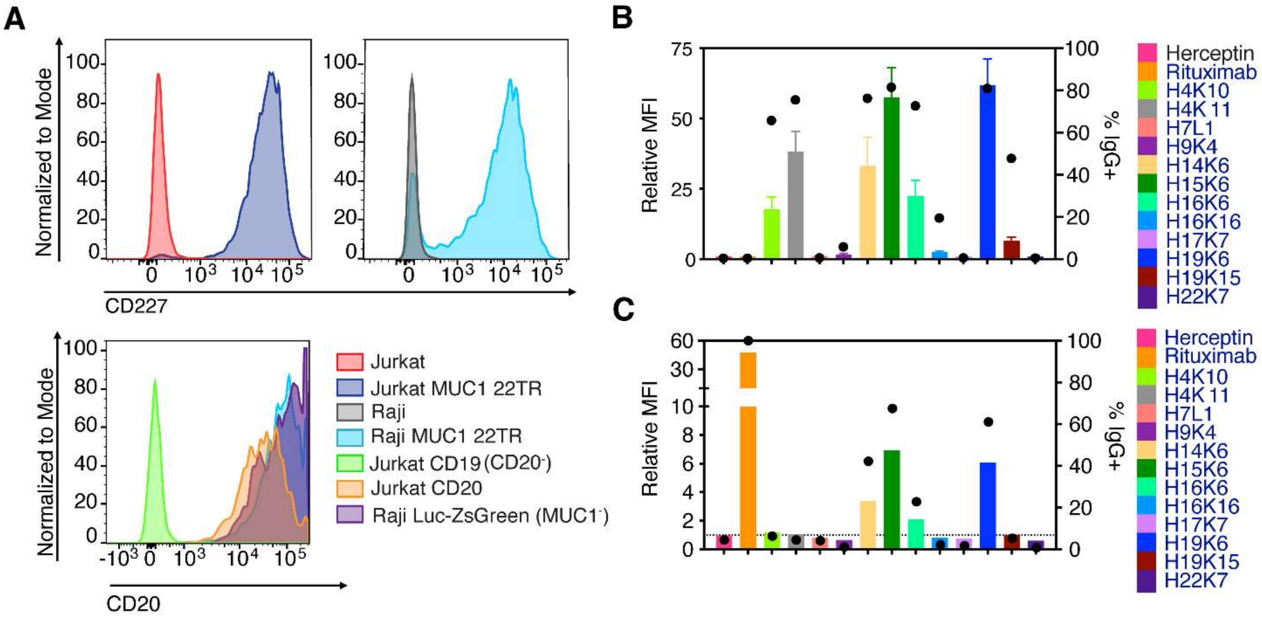

Figure S2. Binding of the human anti-MUC1 mAbs and control antibodies on different target cells. A. Jurkat and Raji cell lines were transduced with MUC1 22TR and stained with mouse anti-MUC1 antibody anti-CD227 (HMPV) to measure total surface MUC1 expression. Jurkat cells were separately transduced with a lentivirus expressing CD20 and stained with rituximab. Jurkat cells transduced with CD19 served as negative controls for rituximab staining. Raji cells endogenously express CD20. **B** and **C.** Jurkat MUC1 22TR (B) and Raji MUC1 22TR (C) were stained with the human mAbs and then with fluorescently-labeled goat anti-human IgG secondary. Relative MFI represents the geometric mean fluorescence intensity (MFI) that was normalized to the negative control Herceptin. % IgG+ shown as black dots. Bars represents the mean +/- SEM for Jurkat MUC1 22TR cells (two replicates each from two independent experiments) or the mean values from an individual Raji MUC1 22TR experiment.

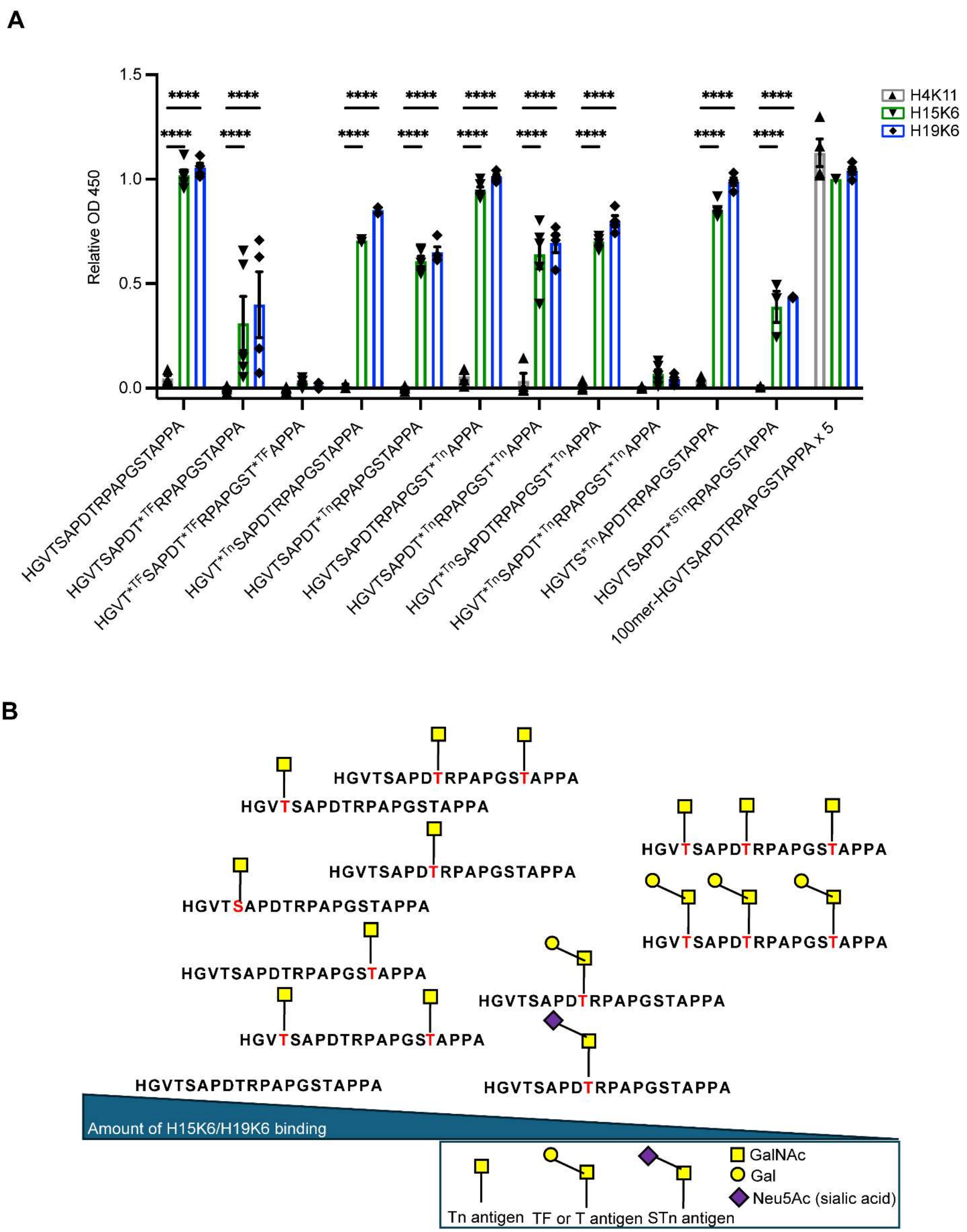

Figure S3. Glycosylation of select serine and threonine residues within the MUC1 tandem repeat can alter anti-human MUC1 IgG binding. A. MUC1 20-mer peptide and glycopeptide sequences were used to coat duplicate wells overnight and then anti-human MUC1 antibody binding was measured by ELISA. Control 100-mer MUC1 sequence was used to provide an antigen that contained epitopes that span the 20-mer sequence. The OD450 of control BSA-only wells was subtracted and resulting OD450 values were normalized to the amount of binding in the H15K6 100-mer well. Each data point represents the average of duplicates. Bars represent mean +/- SEM and n=3 independent experiments using multiple mAb lots are depicted. **** $p<0.0001$ by two-way ANOVA with Šídák's for multiple comparisons. **B.** Ranking binding of H15K6 and H19K6 to individual MUC1 peptide and glycopeptide sequences reveals which sugars and locations are the most permissive and which are the most disruptive to antibody binding.

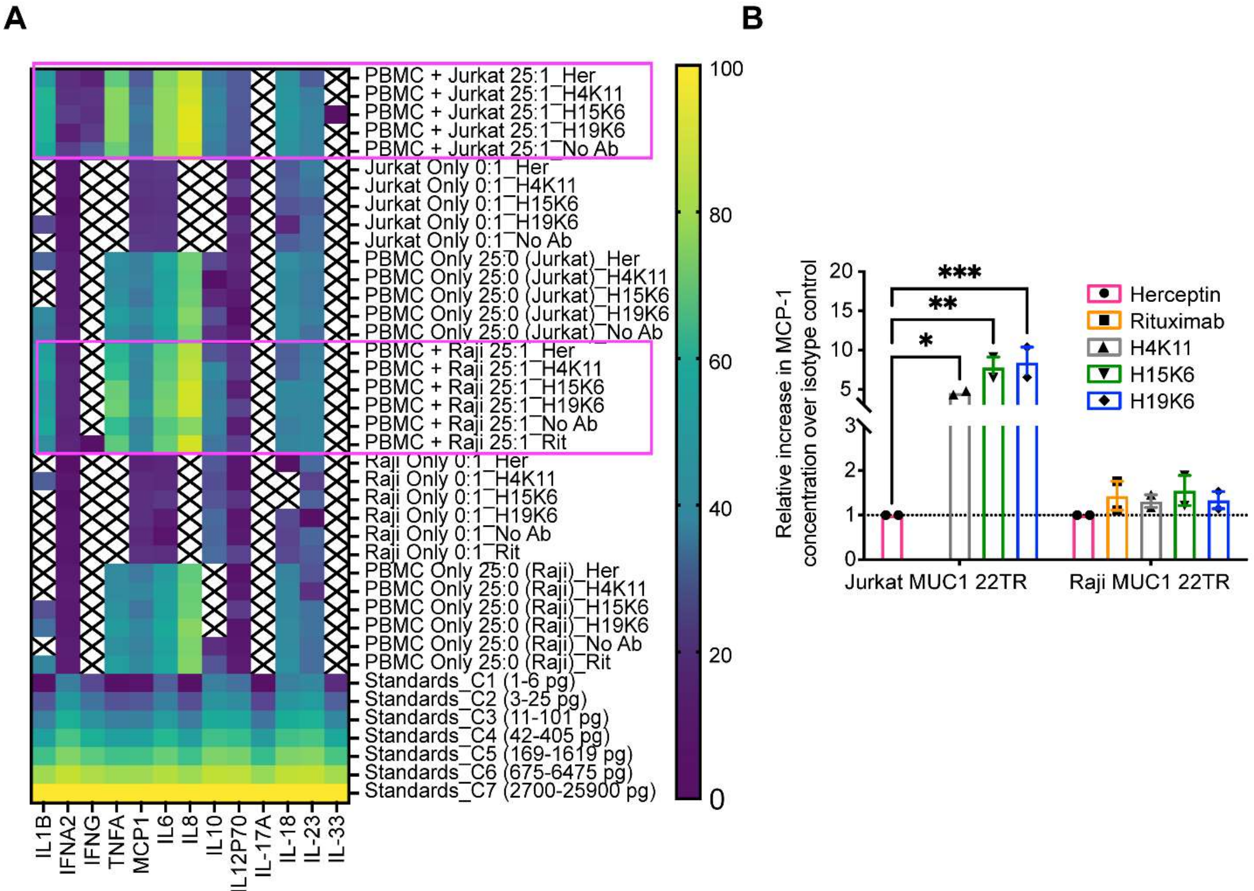

Figure S4. Antibody-mediated cytokine release by PBMCs co-cultured with Raji MUC1 22TR and Jurkat MUC1 22TR in the presence of indicated antibodies. A. Heatmap of normalized values of picogram quantities of 13 cytokines in the supernatants of the cells cultured for the assay. Each value comprises the average of two independent experiments. Co-culture data plotted relative to control wells with isotype Herceptin human IgG antibodies plotted in Figure 2B. **B.** ELISA to measure MCP-1 in the supernatants of the same co-cultures used for the LEGENDplex assay. Each dot represents the value from one experiment, which was each normalized to the amount of cytokine present in the Herceptin condition. Analyses made by two-way ANOVA with Dunnett's for pairwise comparisons *$p<0.05$, **$p<0.01$, ***$p<0.001$.

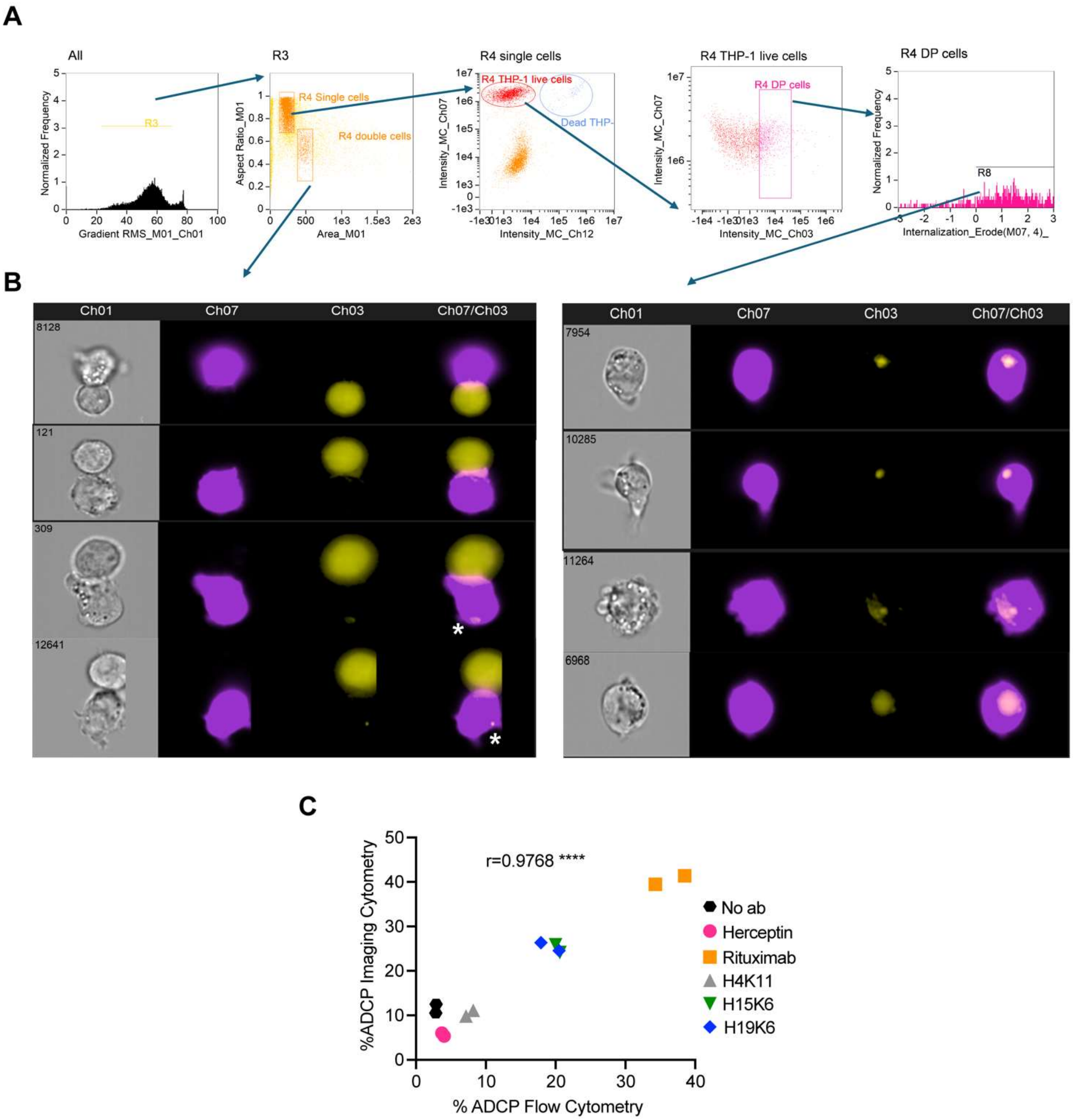

Figure S5. Imaging cytometry confirms human anti-MUC1 mAbs mediate ADCP. A. Gating strategy to select individual THP-1 effector cells (Ch07, purple) vs doublets of THP-1 cells with themselves and other target cells to review images. **B.** Four representative images from the region with events having more area that contained a majority of doublets (Left) and four representative images of events from the region of those with less area. In both panels, events were selected that were high in intensity for CellTrace Yellow, a membrane dye used to label target Raji MUC1 22TR cells (Ch03). Events with a positive internalization score were additionally used to filter images shown in the right panel. (*) highlights images that depict THP-1 cells that additionally contained smaller fragments of CellTrace Yellow. **C.** Assessments of ADCP by imaging cytometry and conventional flow cytometry are highly correlated (Pearson r=0.968, ****p<0.0001). % ADCP by imaging cytometry was calculated by taking the number of events in gate R8 (internalization score positive events) of smaller area (singlet) live THP-1 cells that had some CellTrace Yellow target cell signal in the same image and dividing it by all live single THP-1 cells. %ADCP by flow cytometry was calculated as described in Figure 4.

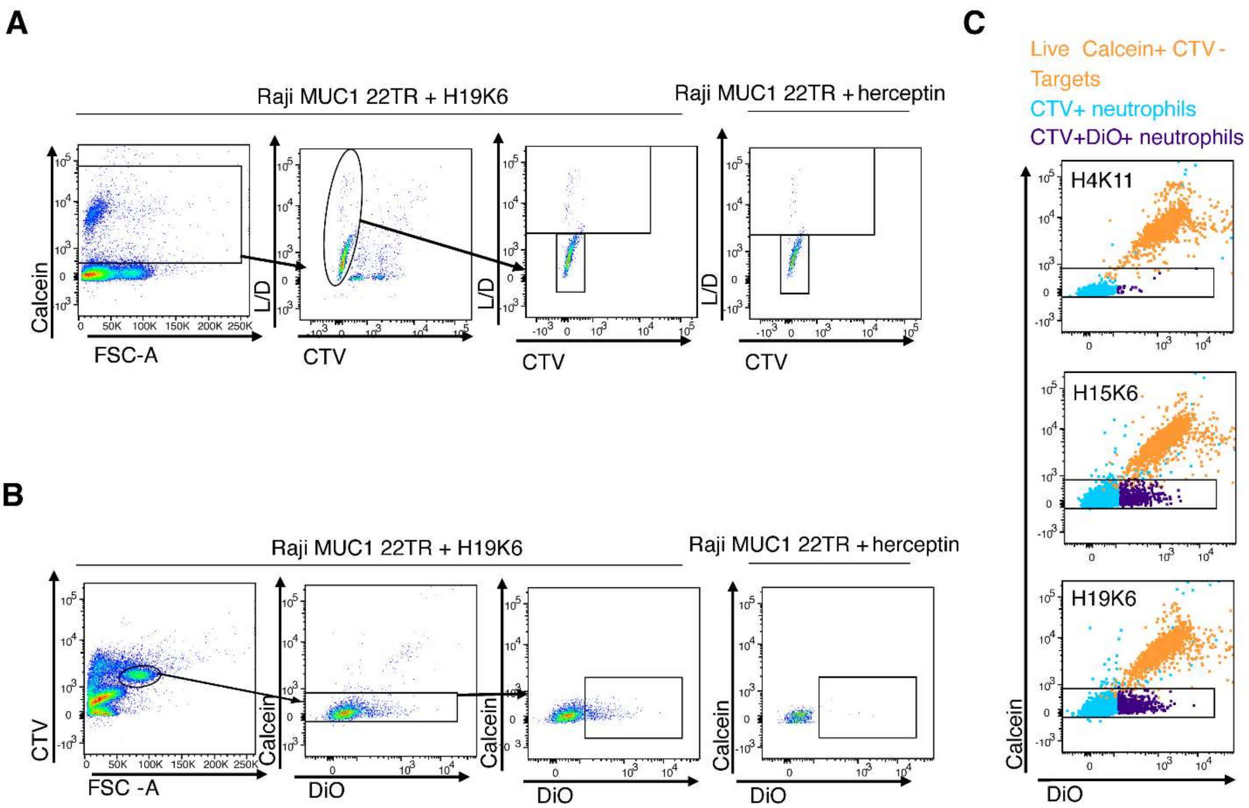

Figure S6. Gating strategy for ADCT experiments. Target Raji MUC1 22TR cells were labeled with Calcein Red-Orange dye to label their cytoplasms and DiO dye to label their surface membranes. Targets were co-cultured with human neutrophils that had been stimulated overnight with G-CSF and IFNgamma. Ghost Dye Red780 was used as a viability stain to discriminate live and dead cells (L/D). **A.** Gating viability of target Raji MUC1 22TR cells after a 4 hour coincubation with stimulated neutrophils and H19K6. Representative plot of viability following co-incubation of the isotype control Herceptin is also shown. **B.** Gating strategy for assessing trogocytosis, by first selecting live neutrophils, and then removing potential doublets with intact target cells based on excluding higher calcein signal. The percentage of DiO+ events of total single neutrophils was used to calculate %ADCT. Raji MUC1 22TR cells after co-culture with H19K6 and control IgG (Herceptin) are shown. **C.** Overlay plots of Calcein+ CellTraceViolet- (CTV-) target Raji MUC1 22TR cells, CTV+ neutrophils and CTV+DiO+ neutrophils in conditions where H4K11, H15K6 and H19K6 were present in co-cultures.

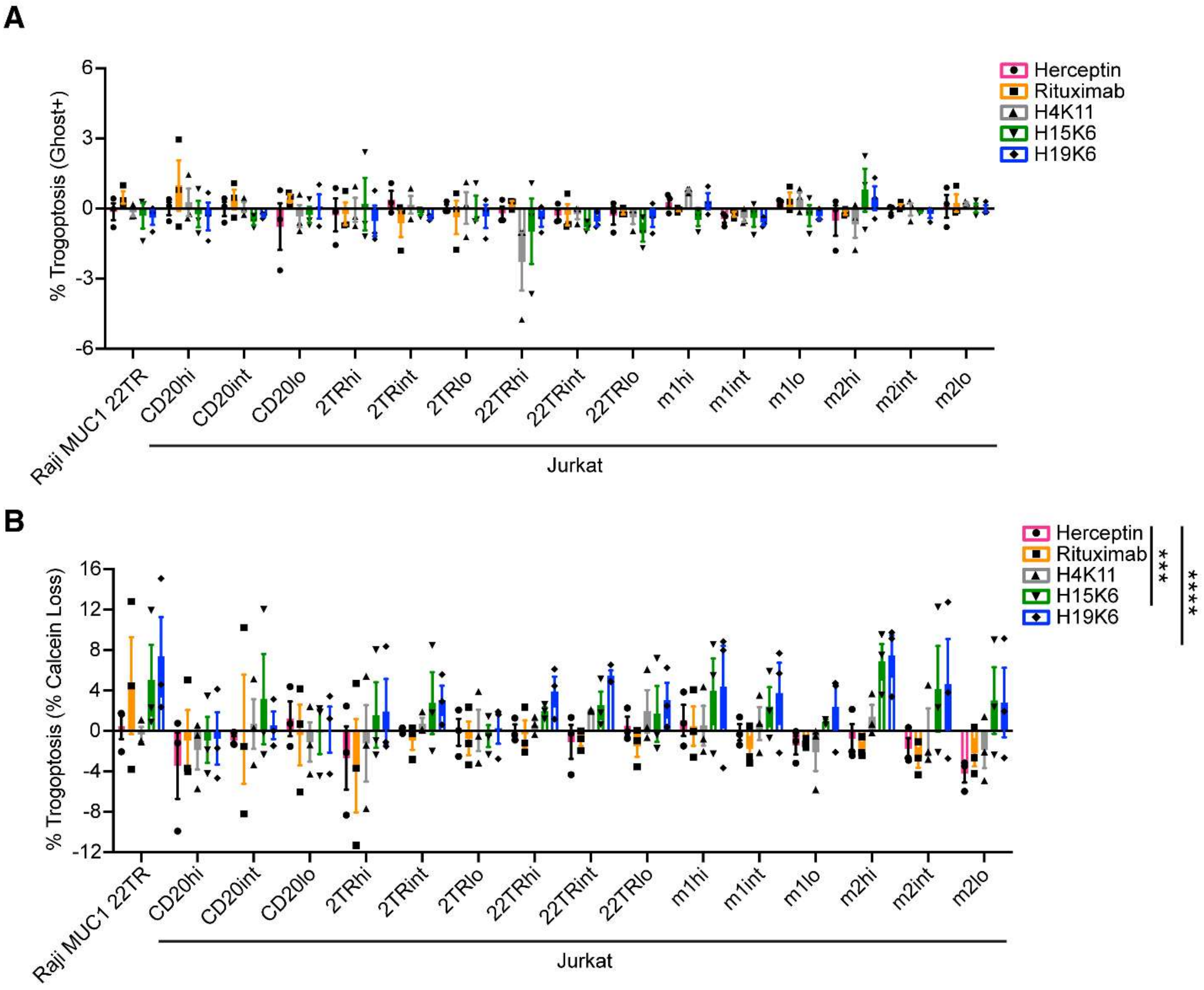

Figure S7. Measurements of cell death during antibody-dependent cellular trogocytosis assays. A. After co-incubation of activated neutrophils, target cells, and human IgG antibodies, cells were stained with the amine-reactive viability dye Ghost Red 780. Percentages of Ghost+ target cells were calculated following the gating scheme shown in Figure S6. All comparisons between groups were not significant by two-way ANOVA with Dunnett's multiple comparisons test. **B.** Trogoptosis measured by calculating the geometric MFI of calcein in Ghost- target cells and normalizing that value to conditions of co-culture where no human IgG antibody was present. Each dot represents the average of duplicates. Bars show the mean +/- SEM of three independent experiments. Groups were compared by two-way ANOVA with Dunnett's multiple comparisons test, ***$p<0.001$, ****$p<0.0001$.

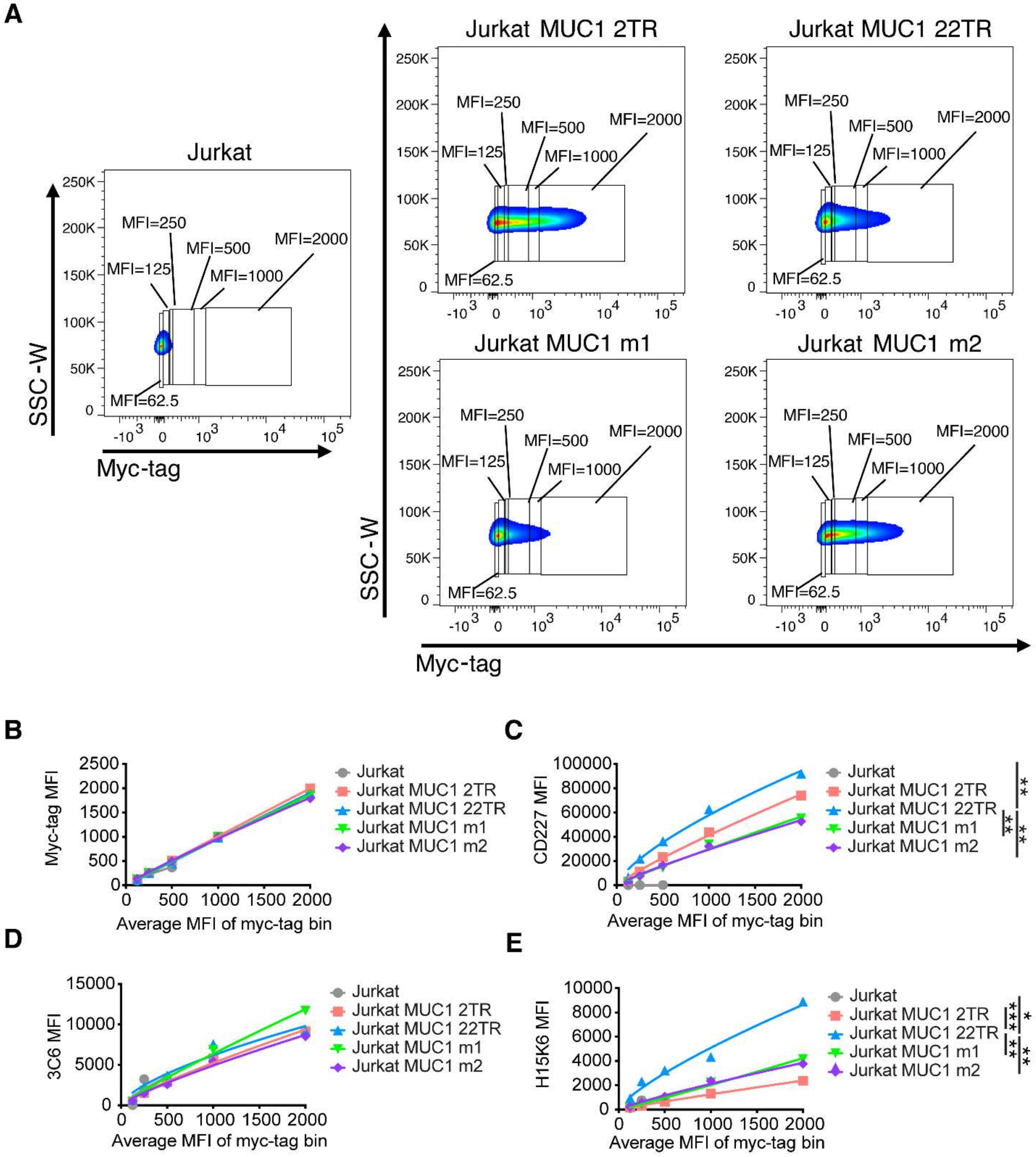

Figure S8. Dual staining with anti-myc and anti-MUC1 antibodies estimates the number of IgGs bound per MUC1 variant molecule. **A.** Cells stained with anti-myc mAbs and anti-MUC1 antibodies were binned into gates that had defined average mean fluorescent intensities of myc. **B-E.** The mean fluorescent intensities of anti-myc-tag and anti-MUC1 mAbs for each population of Jurkat cells in each myc-tag MFI bin were plotted. **B.** Actual MFI of myc-tag by cell line plotted by original MFIs defined for each myc-tag bin. **C.** CD227 vs myc, **D.** 3C6 vs myc, and **E.** H15K6 vs myc. Two-way ANOVA with Tukey's multiple comparison's test was used to compare antibody staining between cell lines, *p<0.05, **p<0.01, ***p<0.001.

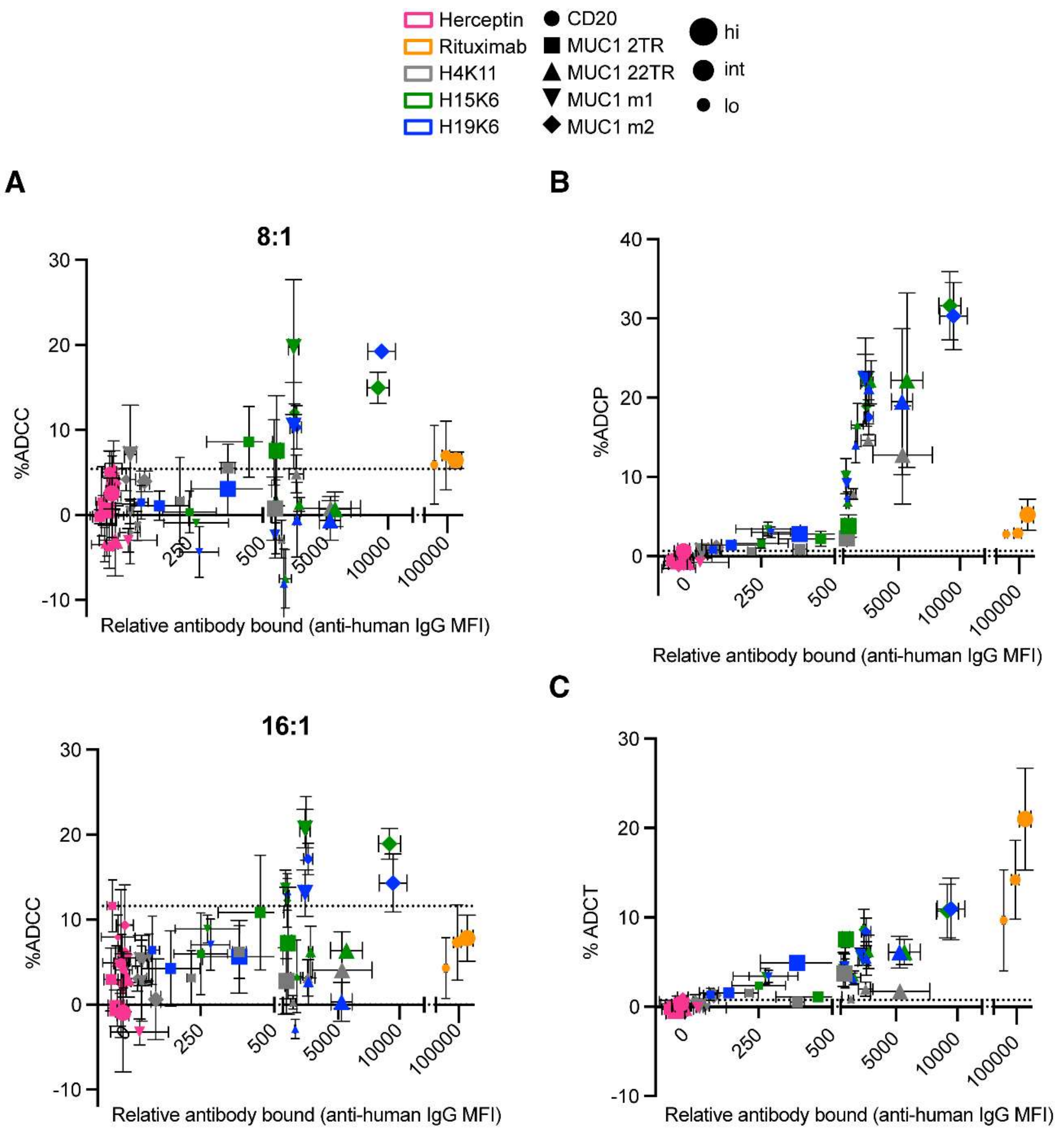

Figure S9. Amount of antibody bound correlates with efficiency of ADCC and ADCP within but not across cell lines possessing various levels of MUC1 variants. Transduced Jurkat cell lines were stained with anti-myc antibodies or rituximab (Jurkat CD20 cells) and sorted for high (hi), intermediate (int) and low (lo) surface expression. Relative differences in expression between hi, int and lo cell lines were maintained over time, allowing for the comparison of differing numbers of antibodies bound to each individual MUC1 variant. Each cell line was incorporated into ADCC (**A**), ADCP (**B**) and ADCT (**C**) assays as described in Figure 8. For ADCC assays, both 8:1 Effector:Target (E:T) and 16:1 E:T ratios were used. Jurkat cells transduced with human CD20 were used with rituximab as positive controls. %ADCC, ADCP and trogocytosis were calculated as in Figures 3-5. ADCT data from Jurkat lines sorted to have high expression are the same as those depicted in Figure 8C. Colored dots represent mean and black error bars represent +/- SEM for average relative MFI and % antibody-mediated function of the average of duplicates in three independent experiments. When taking into account all cell lines at once, there is no significant correlation between amount of antibody bound (average relative MFI) and average %ADCC (r^2=0.04 8:1; r^2=0.005 16:1) or %ADCP (r^2=0.0002), but there is for trogocytosis (r^2=0.59, p<0.0001). Summary of p values for Pearson correlation coefficients examining each cell line independently can be found in Table S2.

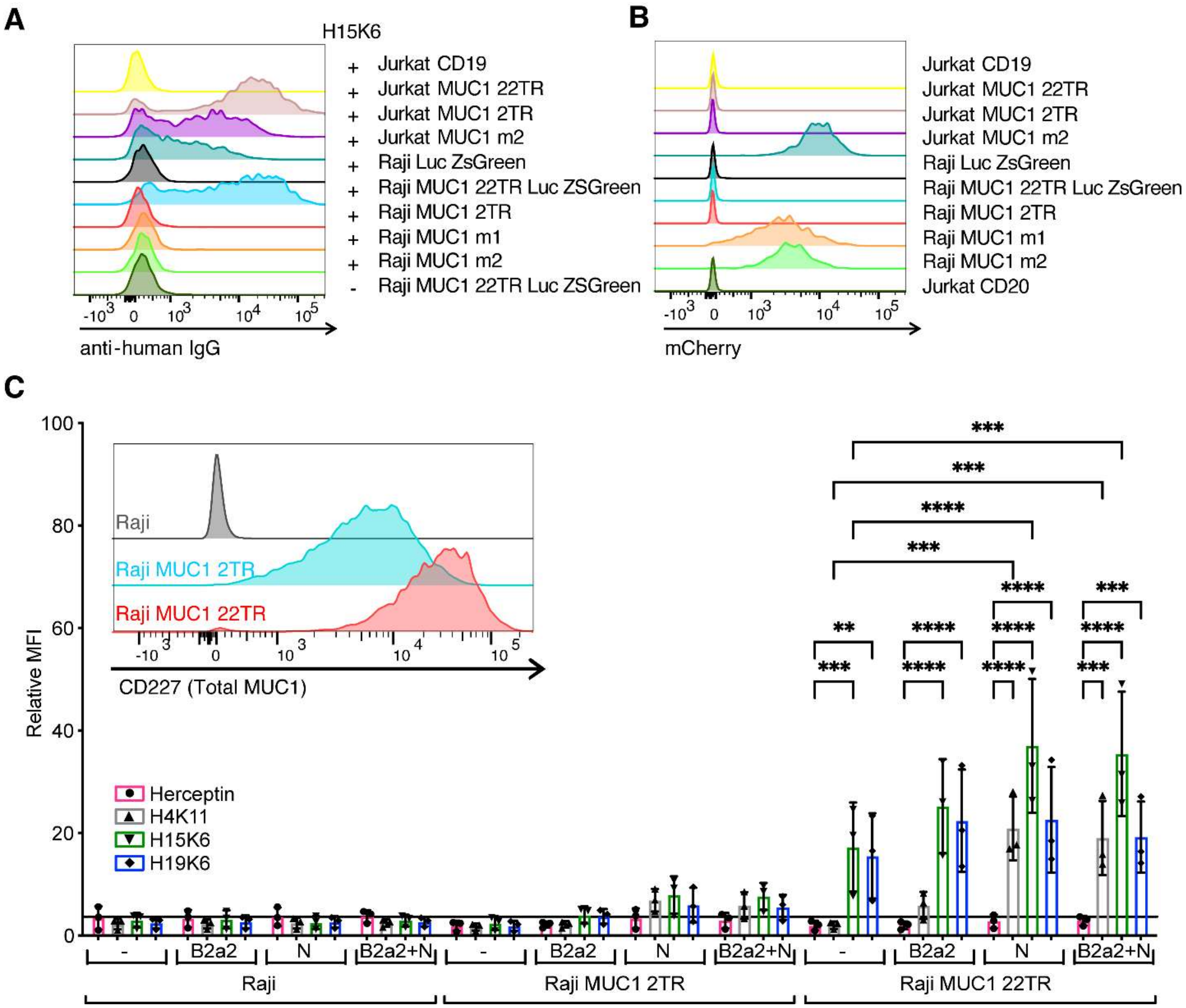

Figure S10. Human anti-MUC1 mAbs do not efficiently stain Raji cells expressing shorter MUC1 variants with 2 tandem repeats.
A. Transduced cell lines were first incubated with H15K6 anti-MUC1 antibody and then anti-human IgG secondary. Raji cells expressing full length 22TR MUC1 are shown with and without H15K6 primary antibody staining as controls. **B.** The level of mCherry reporter expression (present in m1 and m2 constructs) between Jurkat and Raji transduced cell lines. All other cell lines without the reporter construct are also shown for comparison. **C.** Treatment of Raji cell lines with an O-glycosylation inhibitor, Benzyl 2-acetamido-2-deoxy-α-D-galactopyranoside hydrate (B2a2) and neuraminidase (N) to remove sialic acids alone and in combination. Each dot represents one of three independent experiments. Mean +/- SEM displayed. Inset shows total MUC1 levels using the anti-CD227 mAb. Statistical comparisons made by two-way ANOVA with Tukey's multiple comparisons test **p<0.01, ***p<0.001, ****p<0.0001.

Additional Supplemental Material

<u>MUC1 m1 and m2 sequences</u>

Legend

Pink **Signal peptide**

Green **myc-tag**

Blue **CD8α transmembrane**

Grey **(GGGGS)x3 Linker**

Purple **MUC1 20 amino acid tandem repeat**

Orange **MUC1 Cytoplasmic domain**

Red **mCherry**

m1 protein

MALPVTALLLPLALLLHAARPEQKLISEEDLHGVTSAPDTRPAPGSTAPPAHGVTSAPDTRPAPGSTAPPATTTPAPRPPTPAPTI
ASQPLSLRPEACRPAAGGAVHTRGLDFACDIYIWAPLAGTCGVLLLSLVITLYCGGGGSGGGGSGGGGSMVSKGEEDNMAIIKEFM
RFKVHMEGSVNGHEFEIEGEGEGRPYEGTQTAKLKVTKGGPLPFAWDILSPQFMYGSKAYVKHPADIPDYLKLSFPEGFKWERVMN
FEDGGVVTVTQDSSLQDGEFIYKVKLRGTNFPSDGPVMQKKTMGWEASSERMYPEDGALKGEIKQRLKLKDGGHYDAEVKTTYKAK
KPVQLPGAYNVNIKLDITSHNEDYTIVEQYERAEGRHSTGGMDELYK*

m1 DNA sequence

ATGGCTCTCCCAGTCACGGCTCTGCTGCTGCCACTTGCCCTTCTTCTTCACGCCGCCAGACCAGAGCAAAAACTCATATCTGAGGA
AGATTTGCACGGGGTCACCAGCGCACCGGATACCAGACCTGCGCCGGGGTCCACGGCACCCCCTGCTCATGGTGTAACTTCCGCAC
CGGACACACGGCCGGCTCCTGGATCTACAGCACCGCCGGCCACTACTACCCCCGCTCCCAGACCGCCCACCCCGGCTCCTACTATA
GCCTCACAACCGTTGAGTTTGAGACCAGAGGCCTGTCGGCCCGCGGCCGGTGGCGCTGTTCACACGCGAGGACTGGATTTTGCCTG
CGATATTTATATATGGGCTCCACTGGCGGGAACCTGTGGGGTCCTTCTGTTGTCTCTTGTCATAACGCTGTACTGTGGAGGAGGTG
GCAGTGGTGGCGGAGGGAGTGGGGGAGGTGGTTCAATGGTGAGTAAAGGCGAAGAAGACAACATGGCTATCATCAAAGAATTTATG
CGCTTTAAGGTGCATATGGAGGGAAGCGTTAATGGGCACGAATTTGAAATCGAGGGTGAGGGAGAAGGCCGACCCTATGAAGGAAC
GCAGACTGCCAAGCTGAAAGTAACTAAAGGAGGCCCGCTCCCCTTCGCATGGGATATTCTCTCCCCCCAATTTATGTATGGGAGTA
AGGCCTACGTAAAACACCCCGCGGATATCCCCGACTACCTCAAGTTGTCATTCCCGGAAGGTTTCAAGTGGGAGCGAGTTATGAAT
TTTGAAGACGGCGGTGTGGTTACAGTCACCCAAGACTCCAGTTTGCAAGACGGAGAATTTATCTACAAAGTAAAACTGAGAGGTAC
CAACTTTCCGAGTGACGGACCAGTAATGCAGAAGAAAACAATGGGTTGGGAAGCATCTTCAGAGAGAATGTATCCTGAAGACGGTG
CTCTCAAAGGCGAAATCAAACAAAGACTGAAGTTGAAAGATGGAGGCCATTATGATGCAGAAGTAAAGACAACTTACAAAGCTAAG
AAGCCAGTCCAATTGCCTGGGGCTTATAACGTCAATATTAAATTGGACATAACGTCCCATAACGAAGATTACACGATTGTCGAGCA
ATACGAAAGAGCAGAGGGCCGGCATTCAACCGGAGGGATGGATGAACTCTACAAGTGA

m2 protein

MALPVTALLLPLALLLHAARPEQKLISEEDLHGVTSAPDTRPAPGSTAPPAHGVTSAPDTRPAPGSTAPPATTTPAPRPPTPAPTI
ASQPLSLRPEACRPAAGGAVHTRGLDFACDIYIWAPLAGTCGVLLLSLVITLYCGAVCQCRRKNYGQLDIFPARDTYHPMSEYPTY
HTHGRYVPPSSTDRSPYEKVSAGNGGSSLSYTNPAVAATSANLGGGGSGGGGSGGGGSMVSKGEEDNMAIIKEFMRFKVHMEGSVN
GHEFEIEGEGEGRPYEGTQTAKLKVTKGGPLPFAWDILSPQFMYGSKAYVKHPADIPDYLKLSFPEGFKWERVMNFEDGGVVTVTQ
DSSLQDGEFIYKVKLRGTNFPSDGPVMQKKTMGWEASSERMYPEDGALKGEIKQRLKLKDGGHYDAEVKTTYKAKKPVQLPGAYNV
NIKLDITSHNEDYTIVEQYERAEGRHSTGGMDELYK*

m2 DNA sequence

ATGGCCCTGCCCGTAACCGCCCTCCTGTTGCCCCTTGCGTTGTTGCTTCACGCAGCTCGCCCCGAGCAGAAGCTGATTAGTGAAGA
AGATCTCCACGGCGTTACATCTGCACCTGATACCCGACCAGCACCGGGTTCCACCGCGCCACCAGCGCATGGAGTAACGAGCGCCC
CCGATACAAGACCTGCACCTGGATCAACGGCCCCTCCCGCCACGACCACACCGGCACCAAGACCCCCAACACCCGCCCCGACGATA
GCGTCCCAACCCCTCTCTCTCAGGCCGGAAGCGTGCCGCCCCGCAGCGGGGGGAGCTGTGCACACGAGAGGATTGGACTTTGCTTG
TGATATCTATATCTGGGCGCCACTCGCCGGGACGTGCGGTGTTCTCTTGCTGTCCCTGGTGATTACTTTGTATTGTGGAGCGGTGT
GCCAATGCCGGAGAAAGAATTACGGACAGCTCGACATCTTTCCTGCACGCGATACTTACCATCCAATGTCAGAATACCCTACATAC
CACACACACGGTCGGTACGTTCCTCCTTCCTCTACTGATAGGAGCCCCTATGAGAAAGTGAGTGCGGGAAATGGAGGATCATCCTT
GTCTTATACGAATCCGGCCGTTGCCGCTACTTCCGCGAACCTCGGAGGCGGTGGTTCCGGCGGGGGCGGCTCTGGCGGGGGAGGCA
GCATGGTGTCTAAGGGTGAAGAGGATAATATGGCTATTATAAAGGAATTTATGCGGTTTAAGGTACATATGGAGGGCAGCGTCAAT
GGACATGAGTTTGAAATCGAAGGAGAAGGGGAAGGACGCCCTTATGAAGGTACTCAAACTGCAAAATTGAAAGTAACGAAGGGTGG
TCCCCTGCCATTCGCATGGGACATTTTGAGTCCGCAATTCATGTATGGCAGCAAGGCTTATGTCAAGCACCCTGCGGACATACCGG
ACTACTTGAAACTTTCTTTTCCAGAAGGCTTCAAGTGGGAGCGGGTAATGAATTTTGAGGATGGTGGTGTTGTAACCGTCACTCAA
GACTCTTCTCTCCAGGACGGAGAGTTTATATATAAGGTGAAGCTTAGAGGCACGAACTTTCCATCTGATGGGCCAGTAATGCAGAA
AAAAACTATGGGCTGGGAGGCATCTAGTGAGAGAATGTATCCGGAGGATGGCGCGTTGAAAGGGGAAATCAAGCAAAGACTTAAAC
TTAAAGATGGTGGCCACTATGATGCTGAGGTCAAAACAACTTACAAAGCGAAAAAACCTGTTCAATTGCCCGGTGCTTACAACGTC
AATATCAAACTCGATATTACCTCACACAATGAGGACTACACTATCGTCGAACAGTATGAACGGGCTGAAGGACGGCACTCCACCGG
AGGCATGGATGAACTCTATAAATAG

Supplementary Material References:

1. Cascio, S.; Zhang, L.; Finn, O.J. MUC1 Protein Expression in Tumor Cells Regulates Transcription of Proinflammatory Cytokines by Forming a Complex with Nuclear Factor-κB P65 and Binding to Cytokine Promoters: IMPORTANCE OF EXTRACELLULAR DOMAIN *. *J. Biol. Chem.* **2011**, *286*, 42248–42256, doi:10.1074/jbc.M111.297630.
2. Lohmueller, J.J.; Ham, J.D.; Kvorjak, M.; Finn, O.J. mSA2 Affinity-Enhanced Biotin-Binding CAR T Cells for Universal Tumor Targeting. *Oncoimmunology* **2017**, *7*, e1368604, doi:10.1080/2162402X.2017.1368604.
3. Morsut, L.; Roybal, K.T.; Xiong, X.; Gordley, R.M.; Coyle, S.M.; Thomson, M.; Lim, W.A. Engineering Customized Cell Sensing and Response Behaviors Using Synthetic Notch Receptors. *Cell* **2016**, *164*, 780–791, doi:10.1016/j.cell.2016.01.012.
4. Evan, G.I.; Lewis, G.K.; Ramsay, G.; Bishop, J.M. Isolation of Monoclonal Antibodies Specific for Human C-Myc Proto-Oncogene Product. *Mol. Cell. Biol.* **1985**, *5*, 3610–3616, doi:10.1128/mcb.5.12.3610-3616.1985.
5. Pasqual, G.; Chudnovskiy, A.; Tas, J.M.J.; Agudelo, M.; Schweitzer, L.D.; Cui, A.; Hacohen, N.; Victora, G.D. Monitoring T Cell-Dendritic Cell Interactions in Vivo by Intercellular Enzymatic Labelling. *Nature* **2018**, *553*, 496–500, doi:10.1038/nature25442.
6. Shaner, N.C.; Campbell, R.E.; Steinbach, P.A.; Giepmans, B.N.G.; Palmer, A.E.; Tsien, R.Y. Improved Monomeric Red, Orange and Yellow Fluorescent Proteins Derived from Discosoma Sp. Red Fluorescent Protein. *Nat. Biotechnol.* **2004**, *22*, 1567–1572, doi:10.1038/nbt1037.
7. Matz, M.V.; Fradkov, A.F.; Labas, Y.A.; Savitsky, A.P.; Zaraisky, A.G.; Markelov, M.L.; Lukyanov, S.A. Fluorescent Proteins from Nonbioluminescent Anthozoa Species. *Nat. Biotechnol.* **1999**, *17*, 969–973, doi:10.1038/13657.
